# Supplementary material for: SteuerLLM: local specialized large language model for German tax law analysis
Source: Sci Rep. 2026 Jul 10;16:21640. doi: 10.1038/s41598-026-58485-z (PMC13354760; doi:10.1038/s41598-026-58485-z)
Supplement: Supplementary file 1 — Supplementary Material 1 [file 41598_2026_58485_MOESM1_ESM.docx]

**SteuerLLM: Local specialized large language model for German tax law analysis – Supplementary information**

Sebastian Wind (1,2,3), Jeta Sopa (1), Laurin Schmid (1,4), Quirin Jackl (5), Sebastian Kiefer (3), Fei Wu (1), Martin Mayr (1,2), Harald Köstler (2,6), Gerhard Wellein (2), Andreas Maier (1,2), Soroosh Tayebi Arasteh (1,7,8)

1. Pattern Recognition Lab, Friedrich-Alexander-Universität Erlangen-Nürnberg, Erlangen, Germany.
2. Erlangen National High Performance Computing Center, Friedrich-Alexander-Universität Erlangen-Nürnberg, Erlangen, Germany.
3. DATEV eG, Nuremberg, Germany.
4. Bavarian AI Taxation Laboratory, Department of Computer Science, University of Technology Nuremberg, Nuremberg, Germany
5. Chair for Tax Law and Public Law, Friedrich-Alexander-Universität Erlangen-Nürnberg, Nuremberg, Germany.
6. Chair of Computer Science 10, Friedrich-Alexander-Universität Erlangen-Nürnberg, Erlangen, Germany.
7. Lab for AI in Medicine, RWTH Aachen University, Aachen, Germany.
8. Department of Diagnostic and Interventional Radiology, University Hospital RWTH Aachen, Aachen, Germany.

**Correspondence**

Sebastian Wind, MSc ([sebastian.wind@fau.de](mailto:sebastian.wind@fau.de))

Pattern Recognition Lab

Friedrich-Alexander-Universität Erlangen-Nürnberg

Martensstr. 3

91058 Erlangen, Germany


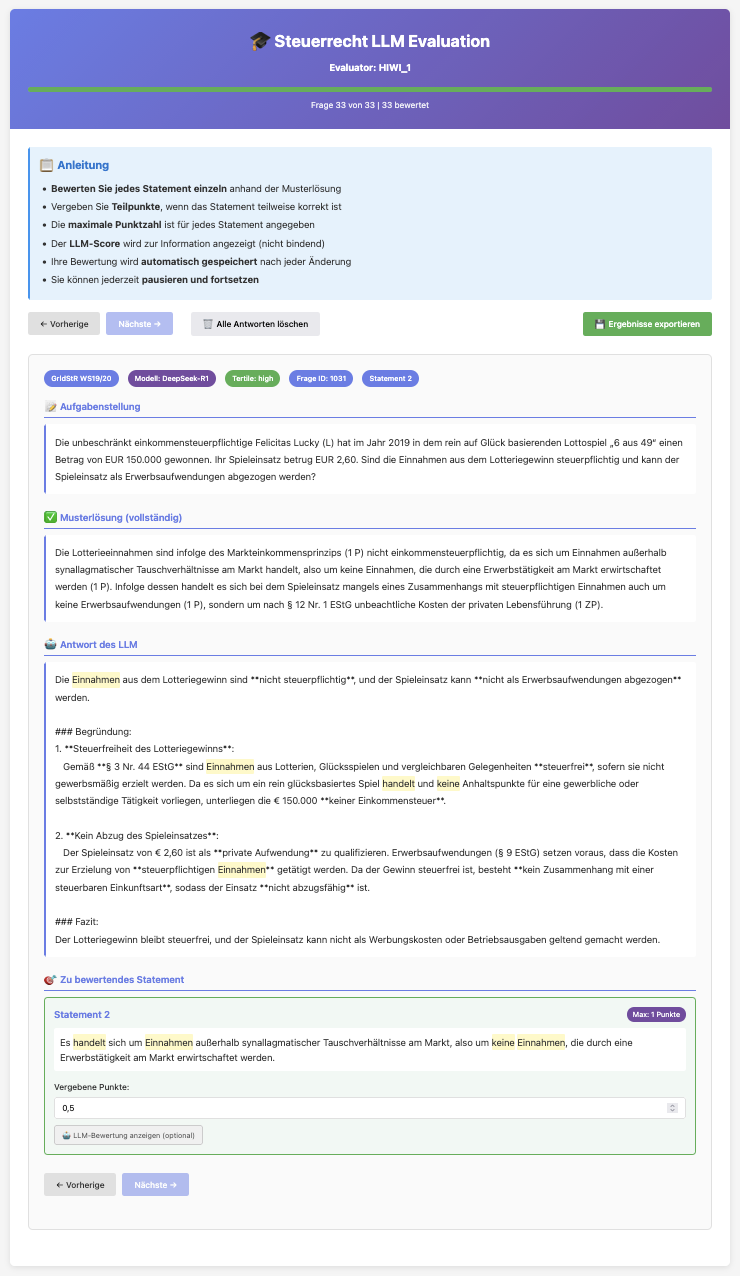


**Supplementary Figure 1:** Web-based human evaluation interface used for statement-level grading of LLM-generated tax law answers. The interface presents the original exam question, the complete reference solution, and the LLM response with highlighted content, followed by an individual examiner statement to be graded. Human evaluators assign partial or full credit according to the original examination rubric, with automatic logging of scores for subsequent inter-rater reliability and human-LLM agreement analyses.


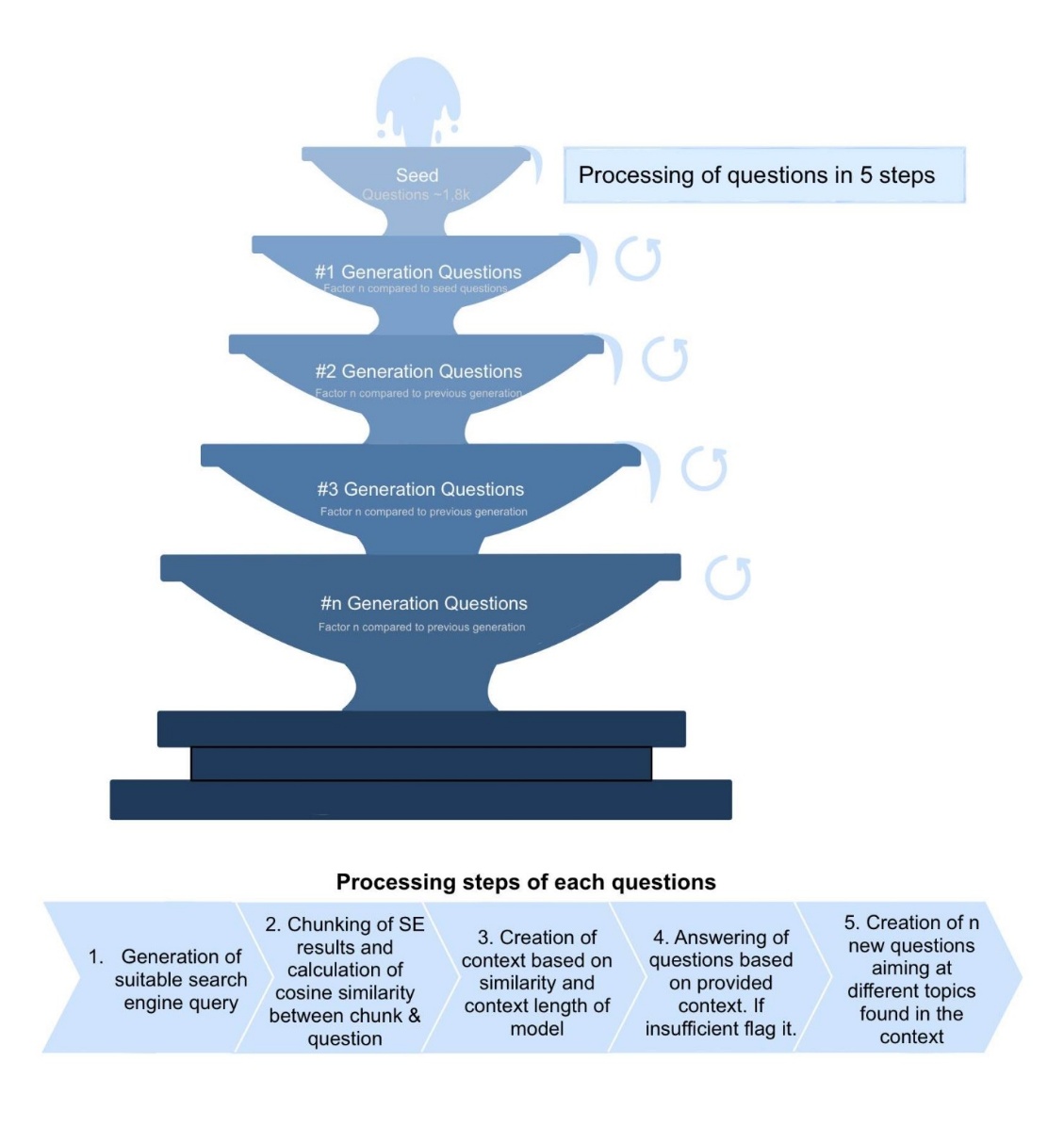


**Supplementary Figure 2:** Iterative structure of the Water Fountain Algorithm for synthetic data generation. The diagram illustrates the multi-stage pipeline used to generate synthetic question-answer pairs for German tax law. Starting from approximately 1,800 authentic examination questions, each generation cycle applies five steps: query generation, retrieval and chunking with similarity scoring, context construction under a fixed context length, answer generation with explicit insufficiency flagging, and creation of multiple new thematically diversified questions. Each layer corresponds to one generation, leading to progressive dataset expansion and increasing topical diversity across iterations.

**Supplementary Table 1:** Subtopic-level composition of the SteuerEx benchmark within selected tax law categories. The table reports the distribution of questions and maximum achievable points across statutory subtopics within individual examinations. Subtopics reflect dominant doctrinal areas covered by each exam and are shown to illustrate internal heterogeneity within benchmark categories. Each question is assigned to exactly one subtopic and counted once. This breakdown is provided for transparency and to contextualize differences in point weighting and difficulty across categories; it is not used directly in model evaluation or statistical analysis.

| Category | Exam name | Subtopic (official German name) | Subtopic (English translation) | Total questions [n] | Maximum achievable points |
| --- | --- | --- | --- | --- | --- |
| Corporate tax (Unternehmensbesteuerung) | UnternehmenSt SS18 | Gewerbesteuer | Trade tax | 1 | 12.0 |
|  |  | Körperschaftsteuer | Corporate income tax | 4 | 36.0 |
|  |  | Umwandlungssteuer | Reorganization tax | 2 | 6.0 |
|  | UnternehmenSt SS19 | Gewerbesteuer | Trade tax | 1 | 13.0 |
|  |  | Körperschaftsteuer | Corporate income tax | 6 | 34.0 |
|  |  | Umwandlungssteuer | Reorganization tax | 1 | 6.0 |
|  | UnternehmenSt SS20 | Gewerbesteuer | Trade tax | 1 | 19.0 |
|  |  | Körperschaftsteuer | Corporate income tax | 5 | 30.0 |
|  |  | Umwandlungssteuer | Reorganization tax | 1 | 7.0 |
|  | UnternehmenSt SS22 | Gewerbesteuer | Trade tax | 1 | 10.5 |
|  |  | Körperschaftsteuer | Corporate income tax | 10 | 36.0 |
|  |  | Umwandlungssteuer | Reorganization tax | 3 | 6.0 |
|  | UnternehmenSt SS23 | Körperschaftsteuer | Corporate income tax | 4 | 20.0 |
|  |  | Körperschaft- & Gewerbesteuer | Corporate & trade tax | 3 | 24.0 |
|  |  | Umwandlungssteuer | Reorganization tax | 1 | 2.0 |
| Fundamentals of tax law (Grundlagen des Steuerrechts) | GrldStR WS23/24 | International tax law | International tax law | 1 | 7.0 |
|  |  | Umsatzsteuer | Value-added tax | 4 | 20.0 |

**Supplementary Table 2:** Exam-level composition of SteuerEx and distribution of student examination performance across individual tax law exams. Each exam-semester pair corresponds to one examination included in the SteuerEx category mapping. For each exam, the table reports the number of students (where available), the number of benchmark questions assigned to the category, and the maximum achievable benchmark points contributed by that exam (as used in the SteuerEx evaluation). Student performance is reported as lowest, average, and highest raw scores under the original grading scheme. Categories are mutually exclusive and each benchmark question is assigned to exactly one category. Student performance statistics are unavailable for some benchmark exams (marked N/A) because the student outcome dataset does not contain exam-level score distributions for those specific exam-semester pairs.

| Category | Exam name | Total students [n] | Total questions [n] | Maximum achievable points | Lowest grade (raw points) | Average grade (raw points) | Highest grade (raw points) |
| --- | --- | --- | --- | --- | --- | --- | --- |
| Corporate tax (Unternehmensbesteuerung) | UnternehmenSt SS18 | 80 | 7 | 54.0 | 15.0 | 34.2 | 45.5 |
|  | UnternehmenSt SS19 | 68 | 8 | 53.0 | 14.5 | 31.1 | 44.5 |
|  | UnternehmenSt SS20 | 84 | 7 | 56.0 | 8.5 | 30.6 | 44.0 |
|  | UnternehmenSt SS22 | 86 | 14 | 52.5 | 9.5 | 29.6 | 51.5 |
|  | UnternehmenSt SS23 | 89 | 8 | 46.0 | 0.5 | 30.5 | 62.0 |
| Fiscal code (Abgabenordnung) | AO SS20 | 16 | 2 | 62.0 | 15.5 | 35.6 | 50.0 |
|  | AO WS16/17 | 29 | 1 | 67.0 | 7.0 | 30.1 | 42.5 |
| Fundamentals of tax law (Grundlagen des Steuerrechts) | GrldStR SS21 | 10 | 12 | 57.0 | 5.5 | 23.9 | 37.0 |
|  | GrldStR WS19/20 | 130 | 15 | 58.0 | 6.0 | 29.8 | 52.0 |
|  | GrldStR WS21/22 | N/A | 6 | 50.0 | N/A | N/A | N/A |
|  | GrldStR WS22/23 | 72 | 11 | 49.0 | 9.5 | 29.1 | 45.5 |
|  | GrldStR WS23/24 | 61 | 12 | 55.0 | 11.0 | 25.7 | 44.0 |
| Income tax (Einkommensteuerrecht) | EStR WS19/20 | N/A | 1 | 63.0 | N/A | N/A | N/A |
|  | EStR WS20/21 | 27 | 1 | 66.0 | 34.5 | 46.3 | 57.0 |
|  | EStR WS21/22 | 32 | 2 | 60.0 | 15.5 | 34.5 | 44.5 |
| Taxation of partnerships (Besteuerung von Personengesellschaften) | PersG SS19 | 16 | 4 | 66.0 | 30.5 | 39.7 | 58.0 |
| Value-added tax (Umsatzsteuerrecht) | USt SS21 | 31 | 2 | 50.0 | 16.0 | 30.0 | 45.5 |
|  | USt SS22 | 22 | 2 | 71.0 | 12.5 | 35.5 | 57.5 |

**Supplementary Table 3:** Human evaluation and validation of automated statement-level grading. Summary of the human evaluation study used to assess both inter-rater reliability among human graders and agreement between human judgments and the automated LLM-based evaluator. The table reports sample composition, exclusion counts due to malformed model outputs, and overlap size used for reliability analysis. Human-human agreement is quantified on the overlap subset using the intraclass correlation coefficient (ICC(2,1)) for absolute agreement. Human-LLM agreement is reported using Kendall’s τ rank correlation between averaged human scores and automated evaluator scores, both overall and stratified^1^ by model. Confidence intervals denote 95% bootstrap intervals. N/A indicates metrics not applicable at the model level.

| Metric | Overall | DeepSeek-R1-671B | Llama-3.2-3B-it | SteuerLLM | GPT-4o-mini |
| --- | --- | --- | --- | --- | --- |
| **Sample composition (n = 59)** | | | | | |
| Items with valid human + LLM score [n] | 59 | 13 | 15 | 15 | 16 |
| Items excluded due to malformed model output [n] | 4 | 4 | 0 | 0 | 0 |
| Overlap items graded by all 3 raters [n] | 20 | N/A | N/A | N/A | N/A |
| **Inter-rater reliability (human vs. human; overlap set only n = 20)** | | | | | |
| ICC(2,1) (absolute agreement) | 0.367 | N/A | N/A | N/A | N/A |
| Perfect agreement among all 3 raters [n (%)] | 3 (15.0%) | N/A | N/A | N/A | N/A |
| Mean score range across raters [%] | 37.99 | N/A | N/A | N/A | N/A |
| Mean within-item SD across raters [%] | 21.07 | N/A | N/A | N/A | N/A |
| Mean absolute difference across raters [%] | 15.84 | N/A | N/A | N/A | N/A |
| Kendall’s τ (HIWI_1 vs. HIWI_2) | 0.625 (P=0.0007) | N/A | N/A | N/A | N/A |
| Kendall’s τ (HIWI_1 vs. HIWI_3) | 0.205 (P=0.273) | N/A | N/A | N/A | N/A |
| Kendall’s τ (HIWI_2 vs. HIWI_3) | 0.314 (P=0.087) | N/A | N/A | N/A | N/A |
| **Human vs. automated evaluator agreement (Kendall’s τ)** | | | | | |
| Kendall’s τ | 0.718 | 0.756 | 0.730 | 0.740 | 0.732 |
| 95% CI for τ | [0.599, 0.820] | [0.460, 0.954] | [0.380, 0.957] | [0.455, 0.935] | [0.460, 0.900] |

**Supplementary Table 4:** Comparison of observed points-weighted accuracy and points-constrained bootstrap mean accuracy across evaluated models. The table reports, for each model, the observed overall accuracy on the SteuerEx benchmark, the accuracy obtained from 1,000 points-constrained bootstrap replicates constructed to match the fixed total point budget of 1,035.5 points, and the resulting shift in percentage points. This comparison quantifies the extent to which the constrained resampling design alters model-level performance estimates relative to its observed benchmark outcome.

| Model | Observed accuracy [%] | Bootstrap mean accuracy  [%] | Shift  (pp) |
| --- | --- | --- | --- |
| Open-SteuerLLM | 29.91 | 23.23 | −6.68 |
| DeepSeek-R1-671B | 44.19 | 38.54 | −5.65 |
| GPT-4o-mini | 26.90 | 21.81 | −5.09 |
| SteuerLLM | 32.93 | 28.38 | −4.55 |
| DeepSeek-R1-Distill-Llama-70B | 23.73 | 19.75 | −3.98 |
| Small-SteuerLLM | 20.11 | 16.49 | −3.62 |
| Gemma-3-27B-it | 26.41 | 22.80 | −3.61 |
| Meta-Llama-3-8B-it | 10.76 | 7.63 | −3.13 |
| Mistral-Small-it-2409 | 22.69 | 20.49 | −2.20 |
| Qwen2.5-72B-it | 20.69 | 18.89 | −1.80 |
| Gemma-3-4B-it | 12.63 | 10.91 | −1.72 |
| Ministral-8B-it-2410 | 14.83 | 13.14 | −1.69 |
| Qwen2.5-14B-it | 13.98 | 12.48 | −1.50 |
| Qwen2.5-3B-it | 6.11 | 5.21 | −0.90 |
| Qwen2.5-32B-it | 18.40 | 17.94 | −0.46 |
| Llama-3.2-3B-it | 8.28 | 8.70 | +0.42 |
| Qwen2.5-7B-it | 11.01 | 11.61 | +0.60 |

**Supplementary Table 5:** Overview of data cleaning and filtering steps applied to the synthetic dataset. This table summarizes the exclusion categories applied during the preprocessing of the raw synthetic data. The cleaning process involved removing exact duplicates, excluding flagged answers based on error indicators, and applying context-based filtering to ensure data quality. A total of 120,625 question-answer pairs were removed to improve dataset diversity, validity, and consistency. N/A: not available.

| Category | Instances | Description |
| --- | --- | --- |
| Initial data cleansing | | |
| Exact duplicates removed | 47,555 | Identified and eliminated duplicates and seed questions to avoid redundancy and ensure dataset diversity. |
| Exact flag-based exclusion | | |
| Flagged answers (exact match) | 11,483 | Answers consisting solely of the flag string (e.g., “Es tut mir Leid”) indicating an error or lack of sufficient context. |
| Context-based filtering | | |
| Total excluded (context-based filtering) | 61,587 | Combined exclusions due to flagged partial matches and insufficient retrieved sources. |
| Flagged answers (partial match) | N/A | Responses containing variations of the flag string within the text, signaling insufficient context or improper formatting. |
| Insufficient retrieved sources | N/A | Tuples where fewer than 3 relevant sources were retrieved from SearXNG, leading to likely insufficient context for a valid response. |

**Supplementary Table 6:** Composition and categorization of the final synthetic training dataset. The final dataset used for fine-tuning SteuerLLM consists of 485,092 question-answer pairs, divided into two main groups: Primary Synthetic Generation and Context-Supported Generation. These categories encompass multiple subtypes targeting specific tax domains and task types, including standard advisory questions, article-based prompts, income tax, accounting, and comment-style analytical tasks. This structure should enable targeted model training for domain adaptation and robust generalization across a broad range of tax-related use cases.

| Category | Instances | Description |
| --- | --- | --- |
| Primary synthetic generation | | |
| Standard data | 215,391 | Base dataset covering various tax law issues derived systematically from a tax advisory practitioner. |
| Diversity data | 62,289 | Designed to increase diversity, featuring alternative task types and complex scenarios to improve generalization. |
| Income tax data | 7,286 | Focused on income tax law, covering e.g. the seven types of income (EStG), taxation of self-employment, and deductibility. |
| Article questions | 20,186 | Questions generated on specific legal paragraphs to improve the model’s ability to reference and interpret them. |
| Accounting record questions | 2,700 | Targeting accounting processes, generated using GPT-4o for better performance in financial accounting. |
| Comment questions | 4,378 | Commentary-style questions that analyze and scrutinize tax law articles. |
| Context-supported generation | | |
| Chunk questions | 172,862 | Generated based on retrieved tax law contexts, ensuring questions relate directly to the given context. |

# **Supplementary Table 7:** Component-level ablation: LoRA in place of block expansion. We compare Open-SteuerLLM (block expansion, 28B parameters total, ≈4B added depth-wise) against a LoRA variant trained under matched optimization budget. The LoRA variant adapts all linear projections of the Mistral-Small-2409 base with rank-32 adapters; the trainable-parameter count of the LoRA variant is approximately 1.8 × 10⁸, substantially smaller than the ≈4 × 10⁹ added by block expansion. We deliberately do not parameter-match the two adaptation strategies: doing so would require either large-rank LoRA (which departs from standard PEFT practice) or partial block expansion (which departs from the published SteuerLLM recipe). Instead, we match the optimization budget and the supervision signal so that any difference is attributable to where the additional capacity is placed (low-rank perturbations alongside frozen weights vs. full Transformer layers inserted into the depth).

| Metric | Open-SteuerLLM(SteuerLLM Instruct) | Lora-SteuerLLM | P-value |
| --- | --- | --- | --- |
| **Bootstrap mean** | **23% ± 3** [18-29] | **20% ± 2** [16-23] | **0.0106** |
| **Total points** | **241 / 1035.5** | **204 / 1035.5** | **-** |

# **Supplementary Table 8:** We perform a single complementary analysis. On the same statement-level item basis as the main result (n = 59; identical exclusion of four items from two DeepSeek-R1 responses with malformed output), we recompute Kendall's τ between the automated evaluator and each individual rater over the items that rater graded (HIWI_1: n = 31; HIWI_2: n = 32; HIWI_3: n = 34; the 20 triple-rated items contribute to all three subsets). This isolates the rater-specific signal from the consensus average and tests whether the headline agreement is driven by any one annotator. Per-(rater × model) τ is positive in all 12 cells (n = 6–10 per cell; CIs wide as expected). Agreement with the automated evaluator is significant and positive under every single-rater ground truth (p ≤ 0.003). HIWI_1 and HIWI_2 bracket the aggregate estimate; HIWI_3's lower τ tracks this rater's lower human–human agreement (pairwise τ = 0.205 and 0.314 with HIWI_1 and HIWI_2, respectively) rather than indicating an LLM-specific failure mode. The ranking signal the LLM judge extracts is therefore preserved across raters, supporting the main-text conclusion that model-level rankings, not absolute per-statement scores and are the validated quantity.

| Rater | n | τ | 95% CI | P-value |
| --- | --- | --- | --- | --- |
| HIWI_1 | 31 | 0.665 | [0.465-0.833] | < 10⁻⁴ |
| HIWI_2 | 32 | 0.802 | [0.653-0.924] | < 10⁻⁴ |
| HIWI_3 | 34 | 0.436 | [0.163-0.665] | 0.003 |
| Aggregate (main text) | 59 | 0.718 | [0.599-0.820] | < 10⁻⁴ |

**Supplementary Table 9:** Reproduction-relevant continual-pretraining hyperparameters for SteuerLLM (28B) and the matched LoRA ablation. Both runs train on the same Mistral-Small 24B base and the same FineWeb German tax-filtered corpus, on an identical 8 × 4 H100 cluster (32 GPUs total), with cosine LR schedule, BF16 mixed precision, and an effective global batch size of 256 sequences (≈ 1.05 M tokens / step). The sole controlled point of difference is the architectural intervention, LLaMA-Pro block expansion versus low-rank adapters reported in the row of the same name. Settings absent from this table are unchanged Axolotl defaults; AdamW β₁, β₂ and ε are listed for completeness because both configurations rely on the framework default rather than overriding it.

| Hyperparameter | SteuerLLM (28B) | LoRA ablation |
| --- | --- | --- |
| Base model | Mistral Small 3 (24B), 40 transformer layers | Mistral Small 3 (24B), 40 transformer layers |
| Architectural intervention | LLaMA-Pro block expansion: 8 identity blocks inserted at indices 5, 11, 17, 23, 29, 35, 41, 47 of the 48-layer expanded model; in each new block, down_proj and o_proj are zero-initialized, all other weights copied from the preceding original block | LoRA adapters on q_proj, k_proj, v_proj, o_proj, gate_proj, up_proj, down_proj of every one of the 40 layers; r = 32, α = 16 (α/r = 0.5), dropout = 0.05 |
| Trainable parameters | 8 inserted blocks + embed_tokens + lm_head; remaining 40 base layers frozen | LoRA A/B matrices only; full 24B base, embeddings and lm_head frozen |
| Optimizer | adamw_bnb_8bit (8-bit AdamW, bitsandbytes) | adamw_torch_fused (PyTorch fused AdamW, full-precision states) |
| AdamW (β₁, β₂, ε) | (0.9, 0.999, 1×10⁻⁸) — framework defaults; not overridden | (0.9, 0.999, 1×10⁻⁸) — framework defaults; not overridden |
| Weight decay | 0.1 | 0.1 |
| Learning-rate schedule | cosine; peak LR = 1×10⁻⁵; warmup_ratio = 0.06 | cosine; peak LR = 1×10⁻⁵; warmup_ratio = 0.06 |
| Epochs | 3 | 3 |
| Micro-batch size (per GPU) | 8 | 4 |
| Gradient-accumulation factor | 1 | 2 |
| Data-parallel ranks | 32 (8 nodes × 4 H100) | 32 (8 nodes × 4 H100) |
| Effective global batch size | 256 sequences ≈ 1 048 576 tokens | 256 sequences ≈ 1 048 576 tokens |
| Sequence length / packing | 4 096 tokens; sample_packing + pad_to_sequence_len enabled | 4 096 tokens; sample_packing + pad_to_sequence_len enabled |
| Mixed precision | BF16 (FP8 not used, TF32 disabled) | BF16 (FP8 not used, TF32 disabled) |
| Parameter sharding | DeepSpeed ZeRO-3 (zero3_bf16.json), optimizer, gradient and parameter sharding across all 32 ranks | Plain DDP, 24B base is frozen, so only the small LoRA adapters carry optimizer state per rank |
| Memory / kernel optimizations | gradient_checkpointing = true; FlashAttention-2 | gradient_checkpointing = true; FlashAttention-2 |

Supplementary Note 1: Points-constrained bootstrap and permutation-based inference for the SteuerEx benchmark

The SteuerEx benchmark evaluates large language models (LLMs) on German tax law examination questions that carry heterogeneous maximum point values, ranging from 1 to 67 points per question across 115 questions totaling 1,035.5 available points. The primary performance metric is the points-weighted accuracy: the ratio of total earned points to total available points, expressed as a percentage. This metric reflects the examination conventions under which German tax law proficiency is assessed, where questions of greater complexity carry proportionally greater weight.

Quantifying uncertainty around this metric requires a resampling procedure that respects the heterogeneous point structure of the benchmark. We need two types of analysis. First, we require descriptive uncertainty intervals for each model’s performance, characterizing the range of accuracy values that would be expected under variation in the composition of the examination. Second, we require formal hypothesis tests to determine whether observed pairwise differences between models are statistically significant. We address these tasks with complementary methods: a points-constrained bootstrap for the former and paired permutation tests for the latter. In a standard non-parametric bootstrap, resampling a fixed number of observations is appropriate because each observation contributes equally to the statistic of interest. In our setting, however, each question carries a distinct maximum point. A standard bootstrap would resample a fixed number of questions and consequently produce replicate datasets with varying total point denominators.

To address this, we implement a points-constrained bootstrap^2^. Each of $B = 1,000$ bootstrap replicates is constructed by drawing questions with replacement from the full question pool until the cumulative sum of maximum points equals exactly the target budget $T = 1,035.5$. The algorithm proceeds sequentially: at each step, a question is sampled at random from among those whose maximum point value does not exceed the remaining budget, and the budget is decremented accordingly. When the remaining budget reaches zero, the replicate is complete. Situations in which no feasible draw remains are rare; if encountered, the replicate is restarted. For each replicate b, the points-weighted accuracy is computed as the sum of earned points divided by $T$. The bootstrap distribution of these 1,000 accuracy values is then summarized by its mean, standard deviation, and 2.5th and 97.5th percentiles to form a 95% percentile confidence interval.

For pairwise significance testing, we use paired^3^ permutation tests. For each question, we compute the score difference between two models. Under the null hypothesis of no performance difference, the sign of this difference is arbitrary. We generate 10,000 random sign-flip permutations and compute the permuted test statistic for each. The two-tailed p-value is the proportion of permutations yielding a test statistic at least as extreme as the observed value, with a continuity correction of +1 in both numerator and denominator. All p-values are subsequently adjusted for multiple comparisons using the Benjamini-Hochberg procedure at a false discovery rate^4^ of $\alpha= 0.05$.

These two procedures serve different inferential purposes. The bootstrap summarizes variability under a fixed-point budget resampling scheme, whereas the permutation test quantifies evidence for a difference on the observed paired question outcomes.

The constrained bootstrap has a direct consequence for question inclusion probabilities. In a single replicate, the number of times a 1-point question can be drawn is bounded only by the total budget, up to 1,035 times in principle, whereas a 67-point question can appear at most 15 times before exhausting the budget. Moreover, the sequential nature of the sampling procedure introduces path dependence: early selection of high‑weight items reduces the allowable choices later in the replicate, increasing the relative likelihood that low‑weight questions appear in the final steps.

This shift in inclusion probabilities results in a systematic difference between the bootstrap mean and the observed accuracy. Empirically, most models show a downward shift of the constrained bootstrap mean relative to the observed score, with shifts ranging from −6.7 to +0.6 percentage points. The size of this shift varies by model and can influence pairwise bootstrap differences when two models have different sensitivities to question weight. For example, models such as Qwen2.5-32B-it^5^, Llama-3.2-3B-it^6,7^ and Qwen2.5-3B-it^5^ show bootstrap means that are very close to their observed performance. Larger shifts appear in models such as Open-SteuerLLM, DeepSeek-R1-671B^8^, GPT-4o-mini, and SteuerLLM. A key limitation becomes visible when comparing models with different magnitudes of shift. For example, Open-SteuerLLM shows a larger downward shift than SteuerLLM. Because the shifts differ across models, the gap between their bootstrap means does not always follow the same pattern as the observed difference used in permutation testing.

For these reasons, we interpret constrained-bootstrap intervals as descriptive uncertainty summaries under a fixed-point budget design, and we rely on permutation tests for formal inference about pairwise differences between models. **Supplementary Table 4** reports the observed and constrained-bootstrap means for all models to make the magnitude and direction of these shifts transparent.

Supplementary Note 2: Tax-law question type taxonomy used for synthetic data generation

To enable systematic and scalable generation of high-quality synthetic training data, we defined a taxonomy of tax-law question types that reflect recurring reasoning patterns in German tax examinations and professional tax practice. The taxonomy was developed in collaboration with professional tax advisors and served as a structural guide for prompt design and automated question generation within the Water Fountain Algorithm. The question types are not used as explicit supervision labels during training or evaluation but ensure broad coverage of doctrinal, procedural, and computational reasoning tasks.

The final taxonomy comprises 18 question types:

1. **Classification tasks**
   Assignment of facts, income, or transactions to legally defined tax categories or income types under statutory provisions.
2. **Fill-in-the-blank tasks**
   Completion of missing legal terms, statutory references, or numerical thresholds in tax-law statements or provisions.
3. **Sequence tasks**
   Ordering of procedural steps in tax assessments, filings, audits, or administrative processes.
4. **Text comprehension tasks**
   Interpretation of statutory provisions, administrative guidance, or case law excerpts and explanation of their legal implications.
5. **Argumentation tasks**
   Justification of tax-law positions through structured legal reasoning and reference to applicable norms.
6. **Matching tasks**
   Mapping of tax concepts, income types, or legal consequences to predefined categories or classifications.
7. **Extension tasks**
   Completion of incomplete legal enumerations or factual descriptions based on statutory structure.
8. **Explanatory tasks**
   Structured explanation of tax-law concepts or mechanisms in coherent natural language.
9. **Rule extraction tasks**
   Identification of applicable statutory requirements or conditions from legal texts.
10. **Comparison tasks**
    Systematic comparison of alternative tax treatments, income types, or legal regimes.
11. **Question generation tasks**
    Formulation of new tax-law questions based on a given statutory provision or legal concept.
12. **Translation tasks**
    Reformulation of complex tax-law provisions into simplified or non-technical language.
13. **Comprehension and commentary tasks**
    Analytical discussion of statutory provisions, including contextual interpretation and illustrative examples.
14. **Accounting and booking tasks**
    Derivation of accounting entries and identification of affected accounts in tax-relevant business transactions.
15. **Framework analysis tasks**
    Identification of relevant tax-law and legal conditions governing a broader factual constellation.
16. **Correction tasks**
    Detection and correction of incorrect tax treatments, calculations, or legal assumptions.
17. **Source identification tasks**
    Identification of relevant statutory provisions, administrative guidance, or legal sources applicable to a given scenario.
18. **Supplementary optimization tasks**
    Identification of additional tax planning options or legally permissible optimizations beyond an existing solution.

Representative examples of question-answer pairs generated under these categories are shown in **Supplementary Table 1**. Together, this taxonomy ensures that the synthetic dataset covers a wide spectrum of reasoning demands encountered in German tax law, ranging from statutory interpretation and procedural reasoning to numerical computation and accounting integration.

Supplementary Note 3: Representative examples of synthetic question-answer pairs generated by the Water Fountain Algorithm

To illustrate the diversity, legal grounding, and structural complexity of the synthetic training data produced by the Water Fountain Algorithm, we provide representative examples of generated question-answer pairs across multiple generation categories. These examples are not intended to be exhaustive or statistically representative, but to qualitatively demonstrate how different prompt configurations, contextual retrieval strategies, and task types are reflected in the resulting data. All examples are drawn from the validated synthetic dataset used to train SteuerLLM.

**Chunk-based questions**

This category comprises questions generated from bounded textual contexts assembled via semantic retrieval from authoritative tax-law sources. The example concerns the tax treatment of a leasing special payment (Leasingsonderzahlung) for a business vehicle under the income surplus accounting method. The generated answer demonstrates period allocation over the contract term, explicit reference to relevant case law of the Bundesfinanzhof, numerical computation, and integration with the 1% rule cost cap. This category emphasizes grounded reasoning based on retrieved legal context and realistic numerical detail.

**Income tax questions**

Income tax questions focus on scenario-based application of statutory provisions, often involving valuation, classification, and interaction between different tax regimes. The illustrated example addresses the transfer of a business vehicle into the private assets of a managing director and discusses valuation under §6 Abs. 1 Nr. 4 Satz 2 EStG, alternative assessment methods, and the concept of hidden profit distributions. The generated answer combines doctrinal explanation, statutory citation, and illustrative numerical examples.

**Standard advisory scenarios**

This category reflects broadly framed advisory-style questions typical of professional tax consulting. The example discusses the outsourcing of an IT department into a separate GmbH and analyzes the resulting implications for value-added tax and trade tax. The answer integrates multiple statutory frameworks, including UStG and GewStG, and demonstrates structured legal analysis across corporate boundaries.

**Commentary and interpretation questions**

Commentary questions require interpretive discussion of individual statutory provisions. The example focuses on §382 AO and explains its function as a blanket provision in customs-related administrative offenses. The generated answer contextualizes the norm, outlines its legal scope, and provides practical examples illustrating its application in enforcement scenarios.

**Article-based questions.**

These questions center on detailed interpretation of specific statutory articles, often involving threshold calculations and evidentiary requirements. The example addresses the determination and proof of economic neediness under §53 Nr. 2 AO. The answer demonstrates multi-step reasoning, incorporation of administrative guidance (AEAO), numerical thresholds, and exceptions to documentation requirements.

**Accounting and booking questions**

This category targets the intersection of tax law and accounting practice. The example concerns private use of a company vehicle by a business owner and contrasts the 1% rule with the logbook method. The generated answer includes concrete booking entries, account references, and VAT treatment, reflecting realistic accounting workflows.

**Diversity-oriented questions**

Diversity questions are designed to expand topical and doctrinal coverage, including reorganization tax law and less frequently examined provisions. The example discusses §20 Abs. 2 Satz 5 UmwStG and the prevention of negative acquisition costs in reorganizations. The answer combines conceptual explanation with a numerical illustration to clarify the statutory mechanism.

Across all categories, the examples demonstrate that the Water Fountain Algorithm produces legally grounded, context-aware, and structurally diverse question-answer pairs. Generated answers consistently integrate statutory citations, case law references, numerical reasoning, and structured legal argumentation, closely resembling authentic examination and professional advisory material in German tax law.

Supplementary Note 4: Water Fountain algorithm for synthetic German tax law data generation

Starting from a curated seed set of approximately 1,800 authentic German tax law examination questions, the algorithm iteratively retrieves authoritative legal sources via SearXNG, constructs bounded textual contexts using semantic similarity, generates grounded answers, filters insufficiently supported instances, and produces diversified follow-up questions under a fixed growth factor ($k=3$). This process yields a large, validated dataset of legally grounded German tax law question-answer pairs for domain-specific language model training.

Inputs: seed set Q_0 (≈1,800 authentic exam questions); generator G (Mistral-Large-Instruct-2407); embedding model E (intfloat/multilingual-e5-large); retrieval engine R (SearXNG); growth factor k = 3; context-window limit N; maximum iterations T. Output: validated question–answer pairs D.

Initialize D ← ∅.

For t = 1,…,T: for each q ∈ Q_{t−1}:

(1) form search query from q;

(2) D_q ← R(q);

(3) chunk and embed D_q;

(4) sort chunks c by sim(c, q) (Eq. 1);

(5) aggregate top-k chunks until token budget N is reached (Eq. 2), forming context C_q*;

(6) A_q ← G(C_q*, q) (Eq. 3);

(7) if f(A_q) = 1 (Eq. 4) then discard, else add (q, A_q, C_q*) to D;

(8) generate k thematically diversified follow-up questions Q_q' from C_q*; append Q_q' to Q_t.

Return D after deduplication and quality filtering.

# Supplementary Note 5: Worked examples per tax-law category

# To improve accessibility of the benchmark and to make the partial-credit grading procedure explicit, we provide one representative worked example per category. Each example contains: (i) the original question text as presented to students, (ii) the expert-validated reference solution, (iii) the statement-level decomposition with assigned point values m_{q,i} ∈ [0.5, 13], (iv) the SteuerLLM response generated under the deterministic-decoding configuration described in Methods §“Experimental design”, and (v) the per-statement awarded points a_{q,i} produced by the GPT-4o adjudicator. Examples S7.1–S7.6 cover, in order, corporate tax, fiscal code, fundamentals of tax law, income tax, partnership taxation, and value-added tax.

**Example 5.1 Corporate tax (Unternehmensbesteuerung)**

| Exam code | UnternehmenSt |
| --- | --- |
| Term | SS22 |
| Sub-topic title | Körperschaftsteuer 1 |
| Question ID | 1057 |
| Reference total m_q | 1.0 points |
| SteuerLLM awarded a_q | 1.0 points |
| Partial-credit ratio | 100.0 % |

### **(i) Original question text**

Die Felix Lobrecht GmbH (F-GmbH) hält 8% der Anteile an der Gemischtes Hack GmbH (G-GmbH). Die G-GmbH produziert verschiedene Podcasts und verkauft Merch, wie T-Shirts und Kaffeebecher an Fans über einen Onlineshop. Die Gesellschaft hat ihren Sitz und auch den Ort der Geschäftsleitung in Berlin. Die G-GmbH hatte in den vergangenen Jahren hohe Finanzierungsaufwendungen und hat aufgrund der Pandemie hohe Verluste gemacht. Aus diesem Grund hat die Gesellschaft zum 31.12.2021 einen Verlustvortrag i.H.v. EUR 800.000 und Zinsvorträge i.H.v. EUR 70.000. Die Situation bessert sich jedoch und im Februar 2022 schüttet die G-GmbH ihren Gewinn an die Gesellschafter aus. Auf dem Konto der F-GmbH werden EUR 37.500 gutgeschrieben. Der Steuerberater der F-GmbH rät deren Geschäftsführer, im März 2022 weitere 3% der Anteile an der G-GmbH zu erwerben, da die Gewinnausschüttung dann steuerfrei wäre. Die F-GmbH kommt der Empfehlung allerdings nicht nach.

Ist die G-GmbH in Deutschland körperschaftsteuerpflichtig?

### **(ii) Expert-validated reference solution**

Die G-GmbH ist als Kapitalgesellschaft unbeschränkt körperschaftsteuerpflichtig, § 1 Abs. 1 Nr. 1 KStG, weil sie ihre Geschäftsleitung, § 10 AO, und ihren Sitz, § 11 AO, in Deutschland hat (1 P).

### **(iv) SteuerLLM response (deterministic decoding)**

Ja, die G-GmbH ist in Deutschland körperschaftsteuerpflichtig. Gemäß § 1 Abs. 1 Nr. 1 KStG sind Kapitalgesellschaften, die ihren Sitz oder ihre Geschäftsleitung im Inland haben, unbeschränkt körperschaftsteuerpflichtig. Da die G-GmbH ihren Sitz und den Ort der Geschäftsleitung in Berlin hat, erfüllt sie diese Voraussetzungen.

### **(iii)+(v) Statement-level decomposition and awarded points**

| i | Reference statement (with m_{q,i}) | Awarded a_{q,i} – adjudicator reasoning |
| --- | --- | --- |
| 1 | Die G-GmbH ist als Kapitalgesellschaft unbeschränkt körperschaftsteuerpflichtig, § 1 Abs. 1 Nr. 1 KStG, weil sie ihre Geschäftsleitung, § 10 AO, und ihren Sitz, § 11 AO, in Deutschland hat (1 P).  m_{q,1} = 1 P | 1 / 1 P  Der Prüfling hat den Kerninhalt des Statements korrekt erfasst, indem er die unbeschränkte Körperschaftsteuerpflicht der G-GmbH aufgrund ihres Sitzes und ihrer Geschäftsleitung in Deutschland gemäß § 1 Abs. 1 Nr. 1 KStG erläutert hat. |

Total awarded: Σ a_{q,i} = 1.0 / 1.0 P (100.0 %)

**Example 5.2 Fiscal code (Abgabenordnung)**

| Exam code | AO |
| --- | --- |
| Term | SS20 |
| Sub-topic title | Abgabenordnung 20 |
| Question ID | 1108 |
| Reference total m_q | 13.0 points |
| SteuerLLM awarded a_q | 4.5 points |
| Partial-credit ratio | 34.6 % |

### **(i) Original question text**

Martin Mies (M) erzielte Einkünfte aus Vermietung einer schönen Doppelhaushälfte in München und selbständiger Tätigkeit als Architekt.

Da M trotz mehrfacher Aufforderung für 2014 keine Einkommensteuererklärung abgegeben hatte, erließ das Finanzamt 2015 einen Einkommensteuerbescheid, mit dem es die Einkommensteuer auf 4.500 € festsetzte. Der Bescheid stand unter dem Vorbehalt der Nachprüfung.

Nachdem M später im Jahr 2015 doch noch eine Einkommensteuererklärung für das Jahr 2014 abgegeben hatte, wurde im folgenden Jahr bei ihm eine Außenprüfung für das Jahr 2014 durchgeführt. Daraufhin wurde Anfang 2017 ein für 2014 (formell ordnungsgemäßer) Änderungsbescheid erlassen, in dem die Steuer auf 5.000 € festgesetzt wurde. Der Bescheid, der keine Ausführungen zum Vorbehalt der Nachprüfung enthielt, wurde von M nicht mittels Rechtsbehelfen angegriffen.

Am 17.6.2020 erreichte M ein Brief des Finanzamtes datiert auf und mit Poststempel vom 16.6.2020. Dabei handelte es sich erneut um eine (formell ordnungsgemäße) Änderung des Einkommensteuerbescheids für das Jahr 2014, in dem die Steuer nunmehr auf 7.000 € festgesetzt wurde. Hintergrund dieser Änderung war, dass bei Durchsicht der Steuerakten festgestellt worden war, dass sich die Einkünfte aus Vermietung der Doppelhaushälfte auf 25.000 € und nicht, wie von M grob fahrlässig erklärt, auf 15.000 € beliefen. Auch waren weitere Werbungskosten in Höhe von

5.000 € trotz anfänglicher Mitteilung durch M unberücksichtigt geblieben.

M stand dem Ganzen misstrauisch gegenüber. Immerhin könne es nicht sein, dass der Steuerbescheid nach durchgeführter Außenprüfung erneut geändert werde. Das Finanzamt berief sich demgegenüber auf § 164 AO.

Am 20.7.2020 ging M zum Finanzamt und wollte eine Berichtigung des Steuerbescheids durch eine vom Finanzamt niederzuschreibende Erklärung erwirken. Allerdings wurde ihm dort mitgeteilt, dass dies wegen CoVid-19 nicht möglich sei; er solle doch bitte einen Brief schreiben. Am 25.7.2020 sandte M dann ein Fax mit folgendem Inhalt an das (zuständige) Finanzamt: „Meine sehr geehrten Damen und Herren, hiermit lege ich Beschwerde gegen den Bescheid vom 16.6.2020 ein. <<Unterschrift M>>“.

Als wäre das nicht genug, stellt sich später heraus, dass die Einkünfte des M aus selbstständiger Tätigkeit vom Finanzamt gänzlich unberücksichtigt blieben. Dies resultierte aus dem Umstand, dass die Anlage S (Einkünfte aus selbstständiger Arbeit) nicht eingescannt und deren Daten nicht automatisiert in das elektronische System übernommen wurden. Trotz vorhandener Prüf- und Risikohinweise durch das maschinelle Risikomanagementsystem erfolgte - selbst im Rahmen der Außenprüfung - kein nachträglicher Abgleich mit den eingereichten Steuerunterlagen. Daraufhin erlässt das Finanzamt (erneut) einen Änderungsbescheid und begründet dies mit § 129 S. 1 AO. M ist erbost. Der Fehler des Finanzamts könne ihm doch nach all den Jahren nicht zum Nachteil gereichen.

Kann das Finanzamt den Änderungsbescheid auf Grundlage des § 129 S. 1 AO erlassen?

Hinweis: Gehen Sie bei der Bearbeitung jeweils vereinfachend von einem konstanten Grenzsteuersatz von 30 % aus.

Auf die nachfolgenden Kalendermonate wird hingewiesen. Gesetzliche Feiertage sind entsprechend grau hinterlegt.

Die folgenden Informationen beziehen sich auf die Monatskalender für Juni, Juli und August 2020. Jeder Monat ist tabellarisch mit den Wochentagen von Montag bis Sonntag dargestellt. Bestimmte Tage sind grau hinterlegt, da sie gesetzliche Feiertage sind.Der Juni 2020 beginnt an einem Montag, der 1. Juni ist als Feiertag markiert. Der Monat umfasst 30 Tage, wobei die letzten beiden Tage, der 29. und 30. Juni, auf einen Montag und Dienstag fallen. Der Juli 2020 startet an einem Mittwoch und hat 31 Tage. Es sind keine Feiertage im Kalender markiert. Der August 2020 beginnt an einem Samstag und hat ebenfalls 31 Tage. Der 15. August, ein Samstag, ist als Feiertag gekennzeichnet. Der letzte Tag des Monats, der 31. August, fällt auf einen Montag.

### **(ii) Expert-validated reference solution**

Eine Korrektur nach § 129 S. 1 AO ist möglich, sofern die Voraussetzungen hierfür vorliegen.

I. Keine Festsetzungsverjährung

Im Übrigen könnte bereits Festsetzungsverjährung eingetreten sein, § 169 I 2, 169 II 1 Nr. 2 AO. (1 P)

Jedoch ist die Ablaufhemmung gem. § 171 II 1 AO zu beachten, wonach die Festsetzungsverjährungsfrist nicht vor Ablauf eines Jahres nach Bekanntgabe des (wenn auch rechtswidrigen) Steuerbescheids endet. (1 P)

II. Offenbare Unrichtigkeit

Nach § 129 S. 1 AO kann die Finanzbehörde Schreibfehler, Rechenfehler und ähnliche offenbare Unrichtigkeiten, die beim Erlass des Verwaltungsakts unterlaufen sind, jederzeit berichtigen. Vorliegend könnte die Nichtberücksichtigung der selbstständigen Einkünfte eine ähnliche offenbare Unrichtigkeit gem. § 129 S. 1 AO sein.

Ähnliche offenbare Unrichtigkeiten sind einem Schreib- oder Rechenfehler vergleichbare mechanische Versehen, wie beispielsweise Eingabe- oder Übertragungsfehler, die ebenso mechanisch, d.h. ohne weitere Prüfung, erkannt und berichtigt werden können. (1 P) § 129 S. 1 AO ist jedoch dann nicht anwendbar, wenn die ernsthafte Möglichkeit besteht, dass die Nichtbeachtung einer feststehenden Tatsache auf einer fehlerhaften Tatsachenwürdigung oder gar auf mangelnder Sachverhaltsaufklärung beruht. (1 P) Dabei ist nicht jede versehentlich unberücksichtigte Tatsache mit einer unvollständigen Sachverhaltsermittlung gleichzusetzen. (1 ZP) Dies ist vielmehr erst dann anzunehmen, wenn für die Besteuerung wesentliche Tatsachen nicht durch ein mechanisches Versehen unberücksichtigt geblieben sind, sondern aus Unachtsamkeit bei der Steuerfestsetzung. (1 ZP)

Offenbar ist eine Unrichtigkeit, wenn sie klar auf der Hand liegt, durchschaubar, eindeutig oder augenfällig ist, maW. für jeden objektiven Dritten klar und deutlich als offenbare Unrichtigkeit erkennbar ist. (1 ZP)

Vorliegend beruhte die Nichtberücksichtigung der selbstständigen Einkünfte darauf, dass die Anlage S nicht eingescannt wurde und somit nicht in das elektronische Datenverarbeitungsprogramm übernommen werden konnte. Dieser Fehler war rein grundsätzlich mechanischer Natur und konnte ebenso mechanisch - d.h. ohne weitere Prüfung - berichtigt werden. (1 P) Hierfür hätte die Anlage S lediglich nochmals eingescannt werden müssen. Der Fehler war auch offenbar iSv § 129 S. 1 AO. (1 P)

II. Ausschluss aufgrund § 88 I AO

Vorliegend könnte eine Korrektur über § 129 S. 1 AO jedoch deshalb ausscheiden, weil der Fehler im Bereich der Sachverhaltsermittlungspflicht der Finanzbehörde gem. § 88 I AO liegt. (1 P) Dies ist der Fall, wenn sich die Unachtsamkeit bei der Bearbeitung des Falles häuft und sich dem Sachbearbeiter aufgrund ergangener Prüf- und Risikohinweise Zweifel an der Richtigkeit einer Eingabe aufdrängen müssten und er trotz dieser Zweifel eine weitere Sachverhaltsaufklärung unterlässt. (1 P) In diesem Fall überlagert der Fehler im Bereich der Sachverhaltsermittlung ein ursprünglich mechanisches Versehen. Eine Beurteilung hat nach den Verhältnissen des Einzelfalls und nach Aktenlage zu erfolgen. (1 P)

Vorliegend erfolgte kein Abgleich trotz Prüf- und Risikohinweise und selbst im Rahmen der Außenprüfung nicht. Die Außenprüfung dient gerade der Ermittlung, Prüfung und Beurteilung der Verhältnisse eines Steuerpflichtigen, um die Gleichmäßigkeit der Besteuerung sicherzustellen. (1 P)

III. Ergebnis

Eine Korrektur gem. § 129 S. 1 AO zum Nachteil des M ist nicht möglich.

### **(iv) SteuerLLM response (deterministic decoding)**

Das Finanzamt kann den Änderungsbescheid auf Grundlage des § 129 S. 1 AO erlassen, wenn es sich um eine offenbare Unrichtigkeit handelt. Eine offenbare Unrichtigkeit liegt vor, wenn der Fehler bei Offenlegung des Sachverhalts für jeden unvoreingenommenen Dritten klar und deutlich als offenbare Unrichtigkeit erkennbar ist.

In diesem Fall wurde die Anlage S (Einkünfte aus selbstständiger Arbeit) nicht eingescannt und deren Daten nicht automatisiert in das elektronische System übernommen. Trotz vorhandener Prüf- und Risikohinweise durch das maschinelle Risikomanagementsystem erfolgte - selbst im Rahmen der Außenprüfung - kein nachträglicher Abgleich mit den eingereichten Steuerunterlagen. Dies stellt eine offenbare Unrichtigkeit dar, da der Fehler bei Offenlegung des Sachverhalts für jeden unvoreingenommenen Dritten klar und deutlich als offenbare Unrichtigkeit erkennbar ist.

Daher kann das Finanzamt den Änderungsbescheid auf Grundlage des § 129 S. 1 AO erlassen.

### **(iii)+(v) Statement-level decomposition and awarded points**

| i | Reference statement (with m_{q,i}) | Awarded a_{q,i} – adjudicator reasoning |
| --- | --- | --- |
| 1 | Eine Korrektur nach § 129 S. 1 AO ist möglich, sofern die Voraussetzungen hierfür vorliegen.  I. Keine Festsetzungsverjährung  Im Übrigen könnte bereits Festsetzungsverjährung eingetreten sein, § 169 I 2, 169 II 1 Nr. 2 AO. (1 P)  m_{q,1} = 1 P | 0 / 1 P  Der Prüfling hat die Festsetzungsverjährung nicht erwähnt, daher fehlt ein wesentlicher Teil des Statements. |
| 2 | Jedoch ist die Ablaufhemmung gem. § 171 II 1 AO zu beachten, wonach die Festsetzungsverjährungsfrist nicht vor Ablauf eines Jahres nach Bekanntgabe des (wenn auch rechtswidrigen) Steuerbescheids endet. (1 P)  m_{q,2} = 1 P | 0 / 1 P  Der Prüfling hat die Ablaufhemmung nach § 171 II 1 AO nicht erwähnt. |
| 3 | Offenbare Unrichtigkeit  Nach § 129 S. 1 AO kann die Finanzbehörde Schreibfehler, Rechenfehler und ähnliche offenbare Unrichtigkeiten, die beim Erlass des Verwaltungsakts unterlaufen sind, jederzeit berichtigen. Vorliegend könnte die Nichtberücksichtigung der selbstständigen Einkünfte eine ähnliche offenbare Unrichtigkeit gem. § 129 S. 1 AO sein.  Ähnliche offenbare Unrichtigkeiten sind einem Schreib- oder Rechenfehler vergleichbare mechanische Versehen, wie beispielsweise Eingabe- oder Übertragungsfehler, die ebenso mechanisch, d.h. ohne weitere Prüfung, erkannt und berichtigt werden können. (1 P)  m_{q,3} = 1 P | 1 / 1 P  Der Prüfling hat den Kerninhalt des Statements korrekt erfasst, indem er die Definition einer offenbaren Unrichtigkeit und deren mechanische Natur beschreibt. |
| 4 | § 129 S. 1 AO ist jedoch dann nicht anwendbar, wenn die ernsthafte Möglichkeit besteht, dass die Nichtbeachtung einer feststehenden Tatsache auf einer fehlerhaften Tatsachenwürdigung oder gar auf mangelnder Sachverhaltsaufklärung beruht. (1 P)  m_{q,4} = 1 P | 0 / 1 P  Der Prüfling hat nicht erwähnt, dass § 129 S. 1 AO nicht anwendbar ist, wenn die Möglichkeit besteht, dass der Fehler auf einer fehlerhaften Tatsachenwürdigung oder mangelnder Sachverhaltsaufklärung beruht. |
| 5 | Dabei ist nicht jede versehentlich unberücksichtigte Tatsache mit einer unvollständigen Sachverhaltsermittlung gleichzusetzen. (1 ZP) Dies ist vielmehr erst dann anzunehmen, wenn für die Besteuerung wesentliche Tatsachen nicht durch ein mechanisches Versehen unberücksichtigt geblieben sind, sondern aus Unachtsamkeit bei der Steuerfestsetzung. (1 ZP) Offenbar ist eine Unrichtigkeit, wenn sie klar auf der Hand liegt, durchschaubar, eindeutig oder augenfällig ist, maW. für jeden objektiven Dritten klar und deutlich als offenbare Unrichtigkeit erkennbar ist. (1 ZP) m_{q,5} = 3 P | 1.5 / 3 P  Der Prüfling hat den Kerninhalt des Statements teilweise erfasst, indem er die Definition einer offenbaren Unrichtigkeit wiedergibt, jedoch fehlt die Unterscheidung zwischen mechanischem Versehen und Unachtsamkeit. |
| 6 | Vorliegend beruhte die Nichtberücksichtigung der selbstständigen Einkünfte darauf, dass die Anlage S nicht eingescannt wurde und somit nicht in das elektronische Datenverarbeitungsprogramm übernommen werden konnte. Dieser Fehler war rein grundsätzlich mechanischer Natur und konnte ebenso mechanisch - d.h. ohne weitere Prüfung - berichtigt werden. (1 P) Hierfür hätte die Anlage S lediglich nochmals eingescannt werden müssen. Der Fehler war auch offenbar iSv § 129 S. 1 AO. (1 P)  m_{q,6} = 2 P | 1 / 2 P  Der Prüfling hat den mechanischen Fehler und die Möglichkeit der mechanischen Berichtigung korrekt erfasst, jedoch nicht die Offenbarkeit des Fehlers gemäß § 129 S. 1 AO. |
| 7 | nII. Ausschluss aufgrund § 88 I AO  Vorliegend könnte eine Korrektur über § 129 S. 1 AO jedoch deshalb ausscheiden, weil der Fehler im Bereich der Sachverhaltsermittlungspflicht der Finanzbehörde gem. § 88 I AO liegt. (1 P)  m_{q,7} = 1 P | 0 / 1 P  Der Prüfling hat nicht erkannt, dass die Korrektur aufgrund der Sachverhaltsermittlungspflicht der Finanzbehörde nach § 88 I AO ausgeschlossen sein könnte. |
| 8 | Dies ist der Fall, wenn sich die Unachtsamkeit bei der Bearbeitung des Falles häuft und sich dem Sachbearbeiter aufgrund ergangener Prüf- und Risikohinweise Zweifel an der Richtigkeit einer Eingabe aufdrängen müssten und er trotz dieser Zweifel eine weitere Sachverhaltsaufklärung unterlässt. (1 P) In diesem Fall überlagert der Fehler im Bereich der Sachverhaltsermittlung ein ursprünglich mechanisches Versehen. Eine Beurteilung hat nach den Verhältnissen des Einzelfalls und nach Aktenlage zu erfolgen. (1 P)  m_{q,8} = 2 P | 0 / 2 P  Der Prüfling hat den Kerninhalt des Statements nicht korrekt erfasst, da er die Bedeutung der Prüf- und Risikohinweise und deren Einfluss auf die Sachverhaltsermittlung nicht berücksichtigt hat. |
| 9 | Vorliegend erfolgte kein Abgleich trotz Prüf- und Risikohinweise und selbst im Rahmen der Außenprüfung nicht. Die Außenprüfung dient gerade der Ermittlung, Prüfung und Beurteilung der Verhältnisse eines Steuerpflichtigen, um die Gleichmäßigkeit der Besteuerung sicherzustellen. (1 P)  III. Ergebnis  Eine Korrektur gem. § 129 S. 1 AO zum Nachteil des M ist nicht möglich.  m_{q,9} = 1 P | 1 / 1 P  Der Prüfling hat den Kerninhalt des Statements korrekt erfasst, indem er die fehlende Berücksichtigung trotz Prüf- und Risikohinweisen sowie im Rahmen der Außenprüfung erwähnt hat. |

Total awarded: Σ a_{q,i} = 4.5 / 13.0 P (34.6 %)

**Example 5.3 Fundamentals of tax law (Grundlagen des Steuerrechts)**

| Exam code | GrldStR |
| --- | --- |
| Term | WS19/20 |
| Sub-topic title | Abgabenordnung 11 |
| Question ID | 1035 |
| Reference total m_q | 1.0 points |
| SteuerLLM awarded a_q | 1.0 points |
| Partial-credit ratio | 100.0 % |

### **(i) Original question text**

Wie lautet die Legaldefinition des Begriffes „Steuern“ und wo ist diese geregelt?

### **(ii) Expert-validated reference solution**

Steuern sind nach der Legaldefinition in § 3 Abs. 1 AO Geldleistungen, die nicht eine Geldleistung für eine besondere Leistung darstellen und von einem öffentlich-rechtlichen Gemeinwesen zur Erzielung von Einnahmen allen auferlegt werden, bei denen der Tatbestand zutrifft, an den das Gesetz die Leistungspflicht knüpft; die Erzielung von Einnahmen kann Nebenzweck sein (1 P).

### **(iv) SteuerLLM response (deterministic decoding)**

Die Legaldefinition des Begriffes „Steuern“ ist in § 3 Abs. 1 der Abgabenordnung (AO) geregelt. Dort heißt es:

„Steuern sind Geldleistungen. Sie sind nicht Gegenleistung für eine besondere Leistung. Steuerpflichtiger ist derjenige, der die Steuer schuldet. Steuern werden von einem öffentlich-rechtlichen Gemeinwesen zur Erzielung von Einnahmen allen auferlegt, bei denen der Tatbestand zutrifft, an den das Gesetz die Leistungspflicht knüpft. Die Erzielung von Einnahmen kann Nebenzweck sein.“

Diese Definition umfasst die wesentlichen Merkmale von Steuern, insbesondere dass sie Geldleistungen sind, die nicht als Gegenleistung für eine besondere Leistung erbracht werden, und dass sie von einem öffentlich-rechtlichen Gemeinwesen zur Erzielung von Einnahmen erhoben werden.

### **(iii)+(v) Statement-level decomposition and awarded points**

| i | Reference statement (with m_{q,i}) | Awarded a_{q,i} – adjudicator reasoning |
| --- | --- | --- |
| 1 | Steuern sind nach der Legaldefinition in § 3 Abs. 1 AO Geldleistungen, die nicht eine Geldleistung für eine besondere Leistung darstellen und von einem öffentlich-rechtlichen Gemeinwesen zur Erzielung von Einnahmen allen auferlegt werden, bei denen der Tatbestand zutrifft, an den das Gesetz die Leistungspflicht knüpft; die Erzielung von Einnahmen kann Nebenzweck sein (1 P).  m_{q,1} = 1 P | 1 / 1 P  Der Prüfling hat den Kerninhalt des Statements korrekt erfasst und die wesentlichen Merkmale der Legaldefinition wiedergegeben. |

Total awarded: Σ a_{q,i} = 1.0 / 1.0 P (100.0 %)

**Example 5.4 Income tax (Einkommensteuerrecht)**

| Exam code | EStR |
| --- | --- |
| Term | WS21/22 |
| Sub-topic title | Einkommensteuer 22 |
| Question ID | 1106 |
| Reference total m_q | 16.0 points |
| SteuerLLM awarded a_q | 5.0 points |
| Partial-credit ratio | 31.3 % |

### **(i) Original question text**

Stephan Schmitz wohnt in Salzburg. Er arbeitet dort als Touristenführer und erzielt in 2021 Einnahmen in Höhe von EUR 12.000. Darüber hinaus ist er an einer bolivianischen Kaffee-Plantage beteiligt. Daraus stehen ihm in 2021 Einnahmen in Höhe von EUR 500.000 zu.

Daneben ist er auch noch an der D-AG beteiligt, die ihren Sitz in Düsseldorf hat. Auf Grund dieser Beteiligung werden ihm für 2021 EUR 100.000 auf sein Konto überwiesen. Stephan Schmitz hatte der Bank in 2021 einen Freistellungsauftrag in voller Höhe erteilt. Stephan Schmitz hatte 5,5% der Aktien der D-AG in 2017 für EUR 10.000 erworben. Sein in 2018 verstorbener Onkel vermachte ihm weitere 0,5% der Anteile an der D-AG. Diese hatte der Onkel, wie Stephan Schmitz, ebenfalls in 2017 für EUR 2.000 erworben. Diese Beteiligung war die erste und, auf Grund seines plötzlichen Todes in 2018, auch die einzige Beteiligung, die der Onkel jemals erworben hatte. Im November 2021 muss die D-AG jedoch völlig unerwartet Insolvenz anmelden und auf Grund ihrer desaströsen finanziellen Lage wird die D-AG schließlich am 30.12.2021 aufgelöst.

Aufgabenstellung: Wie hoch sind die Einkünfte des Stephan Schmitz in 2021 in Deutschland?

### **(ii) Expert-validated reference solution**

A) Persönliche Verhältnisse

I. Persönliche Steuerpflicht

Stephan Schmitz (S) ist als natürliche Person (§ 1 BGB) nicht nach § 1 Abs. 1 EStG unbeschränkt einkommensteuerpflichtig, da er weder seinen Wohnsitz (§ 8 AO) noch einen gewöhnlichen Aufenthalt (§ 9 AO) in Deutschland hat. § 1 Abs. 2 EStG ist ebenfalls nicht einschlägig. Ein Antrag nach § 1 Abs. 3 EStG scheidet ebenfalls aus, da die ausländischen Einkünfte weit überwiegen. S ist aber beschränkt steuerpflichtig nach § 1 Abs. 4 EStG, wenn er inländische Einkünfte i.S.d. § 49 EStG hat. (3P)

Sachliche Steuerpflicht

1. Dividende der D-AG, § 20 EStG

Einkünfte aus Kapitalvermögen sind gemäß § 20 Abs. 1 Nr. 1 EStG Dividenden. Gemäß § 49 Abs. 1 Nr. 5 lit. a aa) EStG handelt es sich um inländische Einkünfte. Seine Einnahmen betragen allerdings nicht EUR 100.000, da es sich hierbei um den Nettobetrag nach Abzug der Kapitalerstragsteuer handelt und die einbehaltene Kapitalertragsteuer nicht von den Einkünften abgezogen werden darf, § 12 Nr. 3 EStG. Der Sparer-Pauschbetrag gem. §§ 20 Abs. 9 S. 1, 50 Abs. 1 S. 1 EStG wurde bereits berücksichtigt, da die Bank einen Freistellungsauftrag erhalten hatte. Demnach betragen beläuft sich der Bruttobetrag der Einnahmen auf EUR 99.199/0,75 = EUR 132.265, 33. Wie oben beschrieben, gilt die ESt durch den Steuerabzug als abgegolten, § 50 Abs. 2 S. 1 EStG.

Einkünfte aus Kapitalvermögen gemäß §§ 2 Abs. 1 S. 1 Nr. 5, 20 Abs. 1 Nr. 1, 49 Abs. 1 Nr. 5 lit. a aa) EStG i.H.v. EUR 132.265, 33. (5P)

2. Auflösung der D-AG, § 17 EStG

Es handelt sich hier um Einkünfte i.S.d. §§ 49 Abs. 1 Nr. 2 lit. e aa) i.V.m. § 17 Abs. 1 und 4 EStG, da Schmidt zu mindestens 1% innerhalb der letzten 5 Jahre an der D-AG beteiligt war und die Auflösung einer Kapitalgesellschaft der Veräußerung ihrer Anteile gleichgestellt ist. Auf Grund des Subsidiaritätsprinzips, § 20 Abs. 8 EStG, handelt es sich hierbei um Einkünfte aus Gewerbebetrieb. Veräußerungsgewinn gem. § 17 Abs. 2 S. 1 EStG ist der Veräußerungspreis von EUR 0 abzüglich der AK § 17 Abs. 2 S. 6 EStG i.H.v. EUR 10.000 (für 5,5% des S) und EUR 2.000 (für 0,5% des Onkels). Der Veräußerungsverlust beträgt demnach EUR 12.000. Allerdings ist hier noch § 17 Abs. 2 S. 6 lit. a EStG zu prüfen, da ein Veräußerungsverlust vorliegt und die Anteile unentgeltlich erworben wurden. Da der Onkel nur zu 0,5 Prozent beteiligt war und damit die Voraussetzungen des § 17 Abs. 1 S. 1 EStG bei ihm nicht vorgelegen haben, ist der Veräußerungsverlust insoweit, also i.H.v. EUR 2.000, nicht zu berücksichtigen. Demnach erzielt S hier einen Verlust i.H.v. EUR 10.000. Gem. § 3 Nr. 40 lit. c EStG ist dieser nur zu 60%, also i.H.v. EUR 6.000 zu berücksichtigen.

EK aus Gewerbebetrieb §§ 2 Abs. 1 S. 1 Nr. 2, 17 Abs. 1 und 4, 49 Abs. 1 Nr. 2 lit. e aa) EStG i.H.v. EUR - 6.000. (6P)

Veranlagungsform

Grundsätzlich ist S mit seinen inländischen Einkünften gem. §§ 25, 26 EStG einzeln zu veranlagen. Es findet der Grundtarif nach § 32a Abs. 1 EStG Anwendung. Hinsichtlich der Dividende, die dem Kapitalertragsteuerabzug unterlegen hat („Überweisung auf das Konto“), §§ 43 Abs. 1 Nr. 1, 43a Abs. 1 S. 1 Nr. 1 EStG, § 30 KStG, gilt jedoch, dass die ESt durch diesen Steuerabzug abgegolten ist, § 50 Abs. 2 S. 1 EStG. (2P)

### **(iv) SteuerLLM response (deterministic decoding)**

Um die Einkünfte des Stephan Schmitz in Deutschland für das Jahr 2021 zu ermitteln, müssen wir die verschiedenen Einkunftsarten und deren steuerliche Behandlung in Deutschland berücksichtigen. Hier sind die relevanten Einkünfte und deren steuerliche Behandlung:

1. **Einkünfte aus nichtselbständiger Arbeit (Touristenführer in Salzburg):**

- Einnahmen: EUR 12.000

- Diese Einkünfte sind in Deutschland steuerpflichtig, da Stephan Schmitz in Deutschland wohnt und arbeitet.

2. **Einkünfte aus der Beteiligung an der bolivianischen Kaffee-Plantage:**

- Einnahmen: EUR 500.000

- Diese Einkünfte sind in Deutschland steuerpflichtig, da Stephan Schmitz in Deutschland wohnt. Es handelt sich um Einkünfte aus Kapitalvermögen.

3. **Einkünfte aus der Beteiligung an der D-AG (Sitz in Düsseldorf):**

- Einnahmen: EUR 100.000

- Diese Einkünfte sind ebenfalls in Deutschland steuerpflichtig, da die D-AG ihren Sitz in Deutschland hat und Stephan Schmitz in Deutschland wohnt. Es handelt sich um Einkünfte aus Kapitalvermögen.

4. **Freistellungsauftrag:**

- Stephan Schmitz hatte der Bank einen Freistellungsauftrag in voller Höhe erteilt. Dies bedeutet, dass die Kapitalerträge bis zur Höhe des Sparer-Pauschbetrags (EUR 801 für Ledige) steuerfrei bleiben.

5. **Veräußerungsgewinn aus der Beteiligung an der D-AG:**

- Stephan Schmitz hatte 5,5% der Aktien der D-AG in 2017 für EUR 10.000 erworben.

- Sein Onkel vermachte ihm weitere 0,5% der Anteile an der D-AG, die dieser ebenfalls in 2017 für EUR 2.000 erworben hatte.

- Die D-AG musste im November 2021 Insolvenz anmelden und wurde am 30.12.2021 aufgelöst.

- Der Veräußerungsgewinn aus der Beteiligung an der D-AG ist steuerpflichtig, da die Beteiligung innerhalb der letzten fünf Jahre vor der Veräußerung erworben wurde (§ 17 EStG).

### Berechnung der steuerpflichtigen Einkünfte:

1. **Einkünfte aus nichtselbständiger Arbeit:**

- EUR 12.000

2. **Einkünfte aus der Beteiligung an der bolivianischen Kaffee-Plantage:**

- EUR 500.000

3. **Einkünfte aus der Beteiligung an der D-AG:**

- EUR 100.000

4. **Veräußerungsgewinn aus der Beteiligung an der D-AG:**

- Der Veräußerungsgewinn ist der Unterschied zwischen dem Veräußerungspreis und den Anschaffungskosten.

- Anschaffungskosten für 5,5% der Aktien: EUR 10.000

- Anschaffungskosten für 0,5% der Aktien (Onkel): EUR 2.000

- Gesamtanschaffungskosten: EUR 12.000

- Veräußerungspreis: EUR 0 (da die Aktien wertlos sind)

- Veräußerungsgewinn: EUR 0 - EUR 12.000 = -EUR 12.000 (Veräußerungsverlust)

### Zusammenfassung der steuerpflichtigen Einkünfte:

- Einkünfte aus nichtselbständiger Arbeit: EUR 12.000

- Einkünfte aus der Beteiligung an der bolivianischen Kaffee-Plantage: EUR 500.000

- Einkünfte aus der Beteiligung an der D-AG: EUR 100.000

- Veräußerungsverlust aus der Beteiligung an der D-AG: -EUR 12.000

### Gesamte steuerpflichtige Einkünfte:

- EUR 12.000 (nichtselbständige Arbeit)

- EUR 500.000 (bolivianische Kaffee-Plantage)

- EUR 100.000 (D-AG)

- -EUR 12.000 (Veräußerungsverlust D-AG)

Gesamte steuerpflichtige Einkünfte: EUR 600.000

### Steuerliche Behandlung:

- Die Einkünfte aus nichtselbständiger Arbeit und die Einkünfte aus Kapitalvermögen (bolivianische Kaffee-Plantage und D-AG) sind in Deutschland steuerpflichtig.

- Der Veräußerungsverlust aus der Beteiligung an der D-AG kann mit anderen Einkünften aus Kapitalvermögen verrechnet werden.

### Fazit:

Die steuerpflichtigen Einkünfte des Stephan Schmitz in Deutschland für das Jahr 2021 betragen EUR 600.000.

### **(iii)+(v) Statement-level decomposition and awarded points**

| i | Reference statement (with m_{q,i}) | Awarded a_{q,i} – adjudicator reasoning |
| --- | --- | --- |
| 1 | Persönliche Steuerpflicht  Stephan Schmitz (S) ist als natürliche Person (§ 1 BGB) nicht nach § 1 Abs. 1 EStG unbeschränkt einkommensteuerpflichtig, da er weder seinen Wohnsitz (§ 8 AO) noch einen gewöhnlichen Aufenthalt (§ 9 AO) in Deutschland hat. § 1 Abs. 2 EStG ist ebenfalls nicht einschlägig. Ein Antrag nach § 1 Abs. 3 EStG scheidet ebenfalls aus, da die ausländischen Einkünfte weit überwiegen. S ist aber beschränkt steuerpflichtig nach § 1 Abs. 4 EStG, wenn er inländische Einkünfte i.S.d. § 49 EStG hat. (3P)  m_{q,1} = 3 P | 0 / 3 P  Der Prüfling hat die persönliche Steuerpflicht von Stephan Schmitz nicht korrekt erfasst, da er fälschlicherweise annimmt, dass Schmitz in Deutschland wohnt und unbeschränkt steuerpflichtig ist. |
| 2 | Sachliche Steuerpflicht  1. Dividende der D-AG, § 20 EStG  Einkünfte aus Kapitalvermögen sind gemäß § 20 Abs. 1 Nr. 1 EStG Dividenden. Gemäß § 49 Abs. 1 Nr. 5 lit. a aa) EStG handelt es sich um inländische Einkünfte. Seine Einnahmen betragen allerdings nicht EUR 100.000, da es sich hierbei um den Nettobetrag nach Abzug der Kapitalerstragsteuer handelt und die einbehaltene Kapitalertragsteuer nicht von den Einkünften abgezogen werden darf, § 12 Nr. 3 EStG. Der Sparer-Pauschbetrag gem. §§ 20 Abs. 9 S. 1, 50 Abs. 1 S. 1 EStG wurde bereits berücksichtigt, da die Bank einen Freistellungsauftrag erhalten hatte. Demnach betragen beläuft sich der Bruttobetrag der Einnahmen auf EUR 99.199/0,75 = EUR 132.265, 33. Wie oben beschrieben, gilt die ESt durch den Steuerabzug als abgegolten, § 50 Abs. 2 S. 1 EStG.  Einkünfte aus Kapitalvermögen gemäß §§ 2 Abs. 1 S. 1 Nr. 5, 20 Abs. 1 Nr. 1, 49 Abs. 1 Nr. 5 lit. a aa) EStG i.H.v. EUR 132.265, 33. (5P)  m_{q,2} = 5 P | 2 / 5 P  Der Prüfling hat den Bruttobetrag der Dividende nicht korrekt berechnet und die Abgeltungswirkung der Kapitalertragsteuer nicht erwähnt. |
| 3 | Auflösung der D-AG, § 17 EStG  Es handelt sich hier um Einkünfte i.S.d. §§ 49 Abs. 1 Nr. 2 lit. e aa) i.V.m. § 17 Abs. 1 und 4 EStG, da Schmidt zu mindestens 1% innerhalb der letzten 5 Jahre an der D-AG beteiligt war und die Auflösung einer Kapitalgesellschaft der Veräußerung ihrer Anteile gleichgestellt ist. Auf Grund des Subsidiaritätsprinzips, § 20 Abs. 8 EStG, handelt es sich hierbei um Einkünfte aus Gewerbebetrieb. Veräußerungsgewinn gem. § 17 Abs. 2 S. 1 EStG ist der Veräußerungspreis von EUR 0 abzüglich der AK § 17 Abs. 2 S. 6 EStG i.H.v. EUR 10.000 (für 5,5% des S) und EUR 2.000 (für 0,5% des Onkels). Der Veräußerungsverlust beträgt demnach EUR 12.000. Allerdings ist hier noch § 17 Abs. 2 S. 6 lit. a EStG zu prüfen, da ein Veräußerungsverlust vorliegt und die Anteile unentgeltlich erworben wurden. Da der Onkel nur zu 0,5 Prozent beteiligt war und damit die Voraussetzungen des § 17 Abs. 1 S. 1 EStG bei ihm nicht vorgelegen haben, ist der Veräußerungsverlust insoweit, also i.H.v. EUR 2.000, nicht zu berücksichtigen. Demnach erzielt S hier einen Verlust i.H.v. EUR 10.000. Gem. § 3 Nr. 40 lit. c EStG ist dieser nur zu 60%, also i.H.v. EUR 6.000 zu berücksichtigen.  EK aus Gewerbebetrieb §§ 2 Abs. 1 S. 1 Nr. 2, 17 Abs. 1 und 4, 49 Abs. 1 Nr. 2 lit. e aa) EStG i.H.v. EUR - 6.000. (6P)  m_{q,3} = 6 P | 3 / 6 P  Der Prüfling hat den Veräußerungsverlust korrekt berechnet, jedoch nicht die steuerliche Berücksichtigung des Verlustes gemäß § 3 Nr. 40 lit. c EStG erwähnt. |
| 4 | Veranlagungsform  Grundsätzlich ist S mit seinen inländischen Einkünften gem. §§ 25, 26 EStG einzeln zu veranlagen. Es findet der Grundtarif nach § 32a Abs. 1 EStG Anwendung. Hinsichtlich der Dividende, die dem Kapitalertragsteuerabzug unterlegen hat („Überweisung auf das Konto“), §§ 43 Abs. 1 Nr. 1, 43a Abs. 1 S. 1 Nr. 1 EStG, § 30 KStG, gilt jedoch, dass die ESt durch diesen Steuerabzug abgegolten ist, § 50 Abs. 2 S. 1 EStG. (2P)  m_{q,4} = 2 P | 0 / 2 P  Der Prüfling hat die Veranlagungsform und die Abgeltungswirkung der Kapitalertragsteuer nicht korrekt behandelt. |

Total awarded: Σ a_{q,i} = 5.0 / 16.0 P (31.3 %)

**Example 5.5 Partnership taxation (Besteuerung von Personengesellschaften)**

| Exam code | PersG |
| --- | --- |
| Term | SS19 |
| Sub-topic title | Besteuerung von Personengesellschaften 1 |
| Question ID | 1112 |
| Reference total m_q | 7.0 points |
| SteuerLLM awarded a_q | 1.0 points |
| Partial-credit ratio | 14.3 % |

### **(i) Original question text**

Der Chefkoch Bernd Bioschmatz (B), der seit vielen Jahren ein Sterne-Restaurant in München (Einzelunternehmen) betreibt, möchte mit seiner in Marketingfragen äußerst versierten und zudem auch wohlhabenden Freundin Rita Rich (R) auch beruflich zusammenarbeiten. Beide planen, von B entwickelte Gourmetsaucen herzustellen und deutschlandweit unter Verwendung des Images von B als bekanntem Sternekoch zu vermarkten. Hierzu gründen B und R mit Wirkung zum 31.12.2018 eine OHG. Der Gesellschaftsvertrag entspricht den Vorschriften des HGB. Die formellen Vorschriften für die Gründung der OHG sind erfüllt.

B bringt aus dem Betriebsvermögen seines Einzelunternehmens (Restaurant) ein unbebautes Grundstück (Buchwert EUR 300.000, gemeiner Wert / Teilwert EUR 500.000) in die OHG ein, auf dem später die Produktions- und Lagerhalle der OHG errichtet werden soll. Das Restaurant wird von B weitergeführt. Insbesondere werden die sonstigen Wirtschaftsgüter, wie z.B. die übrigen Betriebsgrundstücke des Restaurants, nicht veräußert oder eingebracht.

R leistet eine Bareinlage in Höhe von EUR 500.000. B und R erhalten beide ein Kapitalkonto von je EUR 500.000. Beide sind am Ergebnis sowie an den stillen Reserven zu je 50% beteiligt.

Ende 2019 beschließt B, seinen Anteil an der OHG aus haftungsrechtlichen Gründen an die B-GmbH zu veräußern, an der er zu 100% beteiligt ist. Er veräußert den OHG-Anteil mit Wirkung zum 31.12.2019 zum gemeinen Wert. Das Kapitalkonto des B in der Gesamthandsbilanz beträgt zu diesem Zeitpunkt unverändert EUR 500.000. Im Laufe des Jahres 2019 haben sich im Gesamthandsvermögen der OHG zusätzliche stille Reserven in Höhe von EUR 60.000 gebildet.

Zu welchen Werten kann das Betriebsvermögen von B in die neu gegründete OHG steuerrechtlich übertragen werden?

Hinweis: Stille Reserven sollen möglichst nicht auf andere Personen übertragen werden.

### **(ii) Expert-validated reference solution**

Steuerrechtlich könnte das UmwStG zur Anwendung gelangen, wenn steuerfunktionale Einheiten übertragen werden. B bringt sein Einzelunternehmen jedoch nicht zur Gänze in die OHG ein. Da das einzelne unbebaute Grundstück keinen Betrieb bzw. Teilbetrieb darstellt, handelt es sich um keinen von § 24 UmwStG begünstigten Vorgang (2 P).

Die Übertragung erfolgt vielmehr nach § 6 Abs. 5 S. 3 Nr. 1 EStG zwingend zum Buchwert von EUR 300.000, da ein einzelnes Wirtschaftsgut gegen Gewährung von Gesellschaftsrechten aus einem Betriebsvermögen des Mitunternehmers in das Gesamthandsvermögen einer Mitunternehmerschaft übertragen wird. Es besteht der Zwang zur Buchwertfortführung; Wahlrechte zum Ansatz eines Zwischenwerts oder des gemeinen Werts bestehen nicht (§ 6 Abs. 5 S. 1 EStG) (2 P).

Da lt. Hinweis keine stillen Reserven von B auf R übertragen werden sollen und zudem die Kapitalverhältnisse in der Gesamthandsbilanz richtig abgebildet werden müssen, erfolgt der Wertansatz des unbebauten Grundstücks in der steuerlichen Gesamthandsbilanz zum 31.12.2018 mit dem gemeinen Wert/Teilwert in Höhe von EUR 500.000 (1 P). Durch die zwingende Buchwertfortführung wurde für B in der Gesamthandsbilanz ein um EUR 200.000 zu hohes Kapitalkonto ausgewiesen. Zur Korrektur ist eine negative Ergänzungsbilanz (Minderkapital EUR 200.000) für B zu bilden (2 P).

### **(iv) SteuerLLM response (deterministic decoding)**

Die steuerrechtliche Übertragung von Betriebsvermögen in eine neu gegründete OHG kann auf verschiedene Weisen erfolgen, wobei die Wahl der Methode von den steuerlichen Zielen und den spezifischen Umständen abhängt. Hier sind die wichtigsten Aspekte und Methoden:

### 1. Einbringung nach § 24 UmwStG

Die Einbringung von Betriebsvermögen in eine Personengesellschaft, wie eine OHG, kann nach § 24 UmwStG erfolgen. Diese Vorschrift ermöglicht es, das Betriebsvermögen steuerneutral zu übertragen, sofern bestimmte Voraussetzungen erfüllt sind.

#### Voraussetzungen:

- **Einbringung eines qualifizierten Einbringungsgegenstandes**: Dies kann ein Betrieb, ein Teilbetrieb oder ein Mitunternehmeranteil sein.

- **Einbringung in eine Personengesellschaft**: Die OHG muss als Personengesellschaft anerkannt sein.

- **Gewährung von Gesellschaftsrechten**: Der Einbringende muss Gesellschaftsrechte erhalten, die seinem Anteil am Betriebsvermögen entsprechen.

#### Wahlrecht:

- **Buchwertansatz**: Das Betriebsvermögen kann mit dem Buchwert angesetzt werden, was eine steuerneutrale Übertragung ermöglicht.

- **Teilwertansatz**: Alternativ kann das Betriebsvermögen mit dem Teilwert angesetzt werden, was zu einer teilweisen Aufdeckung stiller Reserven führt.

- **Zwischenwertansatz**: Es kann auch ein Wert zwischen Buchwert und Teilwert gewählt werden.

### 2. Einbringung nach § 6 Abs. 5 EStG

Eine weitere Möglichkeit ist die Einbringung nach § 6 Abs. 5 EStG, die eine steuerneutrale Übertragung von Betriebsvermögen innerhalb einer Mitunternehmerschaft ermöglicht.

#### Voraussetzungen:

- **Übertragung innerhalb einer Mitunternehmerschaft**: Die Übertragung muss innerhalb einer Mitunternehmerschaft erfolgen.

- **Keine Entgeltlichkeit**: Die Übertragung darf nicht entgeltlich erfolgen.

#### Wahlrecht:

- **Buchwertansatz**: Das Betriebsvermögen kann mit dem Buchwert angesetzt werden, was eine steuerneutrale Übertragung ermöglicht.

### 3. Einbringung nach § 16 EStG

Wenn die Einbringung nicht unter § 24 UmwStG oder § 6 Abs. 5 EStG fällt, kann die Einbringung nach § 16 EStG erfolgen. Dies führt jedoch zur Aufdeckung stiller Reserven und damit zu einer Besteuerung des Veräußerungsgewinns.

#### Voraussetzungen:

- **Veräußerung des Betriebsvermögens**: Die Einbringung wird als Veräußerung des Betriebsvermögens behandelt.

- **Aufdeckung stiller Reserven**: Die stillen Reserven werden aufgedeckt und besteuert.

### 4. Einbringung nach § 20 UmwStG

Wenn die Einbringung in eine Kapitalgesellschaft erfolgt, kann § 20 UmwStG angewendet werden. Dies ermöglicht eine steuerneutrale Übertragung, sofern bestimmte Voraussetzungen erfüllt sind.

#### Voraussetzungen:

- **Einbringung in eine Kapitalgesellschaft**: Die Einbringung muss in eine Kapitalgesellschaft erfolgen.

- **Gewährung von Gesellschaftsrechten**: Der Einbringende muss Gesellschaftsrechte erhalten.

#### Wahlrecht:

- **Buchwertansatz**: Das Betriebsvermögen kann mit dem Buchwert angesetzt werden, was eine steuerneutrale Übertragung ermöglicht.

- **Teilwertansatz**: Alternativ kann das Betriebsvermögen mit dem Teilwert angesetzt werden, was zu einer teilweisen Aufdeckung stiller Reserven führt.

- **Zwischenwertansatz**: Es kann auch ein Wert zwischen Buchwert und Teilwert gewählt werden.

### Fazit

Die steuerrechtliche Übertragung von Betriebsvermögen in eine neu gegründete OHG kann auf verschiedene Weisen erfolgen, wobei die Wahl der Methode von den steuerlichen Zielen und den spezifischen Umständen abhängt. Die Einbringung nach § 24 UmwStG oder § 6 Abs. 5 EStG ermöglicht eine steuerneutrale Übertragung, während die Einbringung nach § 16 EStG zur Aufdeckung stiller Reserven führt. Die Einbringung nach § 20 UmwStG ist relevant, wenn die Einbringung in eine Kapitalgesellschaft erfolgt.

Es ist wichtig, die spezifischen Voraussetzungen und Wahlrechte der jeweiligen Vorschriften zu berücksichtigen, um die steuerlich optimale Lösung zu finden.

### **(iii)+(v) Statement-level decomposition and awarded points**

| i | Reference statement (with m_{q,i}) | Awarded a_{q,i} – adjudicator reasoning |
| --- | --- | --- |
| 1 | Steuerrechtlich könnte das UmwStG zur Anwendung gelangen, wenn steuerfunktionale Einheiten übertragen werden. B bringt sein Einzelunternehmen jedoch nicht zur Gänze in die OHG ein. Da das einzelne unbebaute Grundstück keinen Betrieb bzw. Teilbetrieb darstellt, handelt es sich um keinen von § 24 UmwStG begünstigten Vorgang (2 P).  m_{q,1} = 2 P | 0 / 2 P  Der Prüfling hat nicht explizit erwähnt, dass das einzelne unbebaute Grundstück keinen Betrieb oder Teilbetrieb darstellt und somit nicht von § 24 UmwStG begünstigt ist. |
| 2 | Die Übertragung erfolgt vielmehr nach § 6 Abs. 5 S. 3 Nr. 1 EStG zwingend zum Buchwert von EUR 300.000, da ein einzelnes Wirtschaftsgut gegen Gewährung von Gesellschaftsrechten aus einem Betriebsvermögen des Mitunternehmers in das Gesamthandsvermögen einer Mitunternehmerschaft übertragen wird. Es besteht der Zwang zur Buchwertfortführung; Wahlrechte zum Ansatz eines Zwischenwerts oder des gemeinen Werts bestehen nicht (§ 6 Abs. 5 S. 1 EStG) (2 P).  m_{q,2} = 2 P | 1 / 2 P  Der Prüfling erwähnt § 6 Abs. 5 EStG und den Buchwertansatz, jedoch fehlt der spezifische Bezug auf die Zwangsregelung und die fehlenden Wahlrechte. |
| 3 | Da lt. Hinweis keine stillen Reserven von B auf R übertragen werden sollen und zudem die Kapitalverhältnisse in der Gesamthandsbilanz richtig abgebildet werden müssen, erfolgt der Wertansatz des unbebauten Grundstücks in der steuerlichen Gesamthandsbilanz zum 31.12.2018 mit dem gemeinen Wert/Teilwert in Höhe von EUR 500.000 (1 P).  m_{q,3} = 1 P | 0 / 1 P  Der Prüfling hat das spezifische Statement nicht behandelt, insbesondere fehlt der Bezug auf den Wertansatz des Grundstücks mit dem gemeinen Wert. |
| 4 | Durch die zwingende Buchwertfortführung wurde für B in der Gesamthandsbilanz ein um EUR 200.000 zu hohes Kapitalkonto ausgewiesen. Zur Korrektur ist eine negative Ergänzungsbilanz (Minderkapital EUR 200.000) für B zu bilden (2 P).  m_{q,4} = 2 P | 0 / 2 P  Der Prüfling hat das spezifische Statement über die Korrektur durch eine negative Ergänzungsbilanz nicht behandelt. |

Total awarded: Σ a_{q,i} = 1.0 / 7.0 P (14.3 %)

**Example 5.6 Value-added tax (Umsatzsteuerrecht)**

| Exam code | USt |
| --- | --- |
| Term | SS21 |
| Sub-topic title | Umsatzsteuer 19 |
| Question ID | 1095 |
| Reference total m_q | 10.0 points |
| SteuerLLM awarded a_q | 6.0 points |
| Partial-credit ratio | 60.0 % |

### **(i) Original question text**

Beurteilen Sie die nachfolgenden Sachverhalte in Teil I hinsichtlich ihrer umsatzsteuerlichen Auswirkungen für Gerti Gabler (G) und Heribert Gabler (H) für den Besteuerungszeitraum 2020. Erörtern Sie die steuerlichen Folgen der Sachverhalte in Teil I auf Grundlage des deutschen Umsatzsteuergesetzes. Beantworten Sie Teil II auf Grundlage der Mehrwertsteuersystemrichtlinie.Beschränken Sie sich in Teil II auf die gestellte Aufgabe, beurteilen Sie im Teil II keine Auswirkungen für Gerti oder Heribert.

Für die einzelnen Sachverhalte ist, soweir möglich, insbesondere auf Steuerbarkeit, Steuerpflicht, Bemessungsgrundlage, Steuerentstehung, Steuerschuldnerschaft und den Vorsteuerabzug einzugehen. Die Steuer für steuerpflichtige Umsätze ist zu berechnen, wo dies möglich ist. G und H sind keine Kleinunternehmer. Wo es der Sachverhalt erlaubt, ist auch anzugeben, für welchen Voranmeldungszeitraum die Vorgänge zu berücksichtigen sind. Voranmeldungszeitraum ist der Kalendermonat. Auch für steuerfreie Umsätze sind, wenn möglich, die Entgelte anzugeben. Gehen Sie davon aus, dass alle Akteure mögliche Wahlrechte und Optionsmöglichkeiten in Anspruch genommen haben und diese formgerecht erklärt haben, außer es werden dazu explizit Angaben im Sachverhalt gemacht.

Soweit aus dem Sachverhalt nichts anderes hervorgeht, sind erforderliche ordnungsgemäße Rechnungen mit allen erforderlichen Angaben für den Vorsteuerabzug, sowie Nachweise, Belege und Aufzeichnungen für etwaige Steuerbefreiungen vorhanden. Alle Unternehmer verwenden, sofern nicht ausdrücklich anderweitig im Sachverhalt angegeben, im innergemeinschaftlichen Waren- und Dienstleistungsverkehr die Umsatzsteuer-Identifikationsnummer (USt-Id.Nr.) ihres Heimatlandes, die Geschäftspartnern auch rechtzeitig mitgeteilt wird. Alle nachfolgend erwähnten Staaten gehören der Europäischen Union an.

Gehen Sie davon aus, dass relevante Umsatzschwellen überschritten werden.

Die Änderungen des UStG aufgrund des Corona-Steuerhilfegesetzes bzw. des Zweiten Corona-Steuerhilfegesetzes sind nicht zu berücksichtigen. Insbesondere ist der Steuersatz von 19 % und 7 % sowie der reguläre Steuersatz (19%) für Restaurations- und Verpflegungsdienstleistungen anzunehmen

Bitte beachten Sie außerdem das Urteil des EuGH vom 27. November 2003, Rs. C-497/01, das der Klausur angehängt ist.

Die Clever-Immo GmbH ist Unternehmerin, entsprechende § müssen nicht geprüft werden.

Verkäufer auf Flohmärkten sind regelmäßig keine Unternehmer. Bestimmen Sie die Steuerbarkeit und den Leistungsort/Ort der Steuerbarkeit folgender Umsätze auf Grundlage der MwStSystRL

Die Spiritcoaching AG (S) verspricht ihren gutgläubigen Kunden Lösungen, damit sie Glück und Erfolg im Leben finden. Inzwischen ist aus einer kleinen Gruppe von Anhängern ein riesiges, multinationales Unternehmen geworden. Von ihrem Sitz in den Niederlanden aus vertreibt die S die verschiedensten Produkte und Leistungen nach ganz Europa. Heribert hat zufällig über einen Mieter seiner Frau einen Vertreter der Spiritcoaching AG kennengelernt und ist seitdem überzeugter Kunde: Er hat in 2020 an einem teuren Einstiegskurs, einem noch teureren „1on1 digital lifecoaching“ und letztendlich sogar an einer sündhaft teuren Präsenzveranstaltung in Köln teilgenommen. Bei dem Einstiegskurs handelt es sich um einen digitalen Online-Kurs, bestehend aus aufgezeichneten Lehrvideos und automatisierten, interaktiven Klick-Elementen. Beim „1on1 digital lifecoaching“ handelt es sich um eine ganz persönliche Videokonferenz, in der einer der Gurus vom Sitz in den Niederlanden aus ein persönliches Coaching durchführt. Bei der Präsenzveranstaltung handelt es sich um ein Seminar in einer großen Kölner Hotelhalle, in der die besten Coaches persönlich anreisen und ihre Erkenntnisse teilen.

### **(ii) Expert-validated reference solution**

Die Spiritcoaching AG ist Steuerpflichtiger i.S.d. Art. 9 I MwStSystRL, da sie eine wirtschaftliche Tätigkeit zur Erzielung von Einnahmen betreibt.(1P) Sie erbringt im Rahmen ihres Unternehmens drei separate Dienstleistungen i.S.d. Art. 24 I MwStSystRL (1P) an den H. Der H ins in seiner Rolle als Konsument kein Steuerpflichtiger im Sinne der MwStSystRL.(1P)

1. Einstiegskurs

Es handelt sich um eine elektronisch erbrachte Dienstleistung nach Art. 58 I lit. c MwStSystRL, die an einen nicht-steuerpflichtigen erbracht wurde. (1P) Der Ort der Dienstleistung ist der Ort der Ansässigkeit/ des Wohnsitzes des H, damit in Nürnberg, Deutschland. Der Umsatz wurde gegen Entgelt erbracht und ist damit in Deutschland steuerbar.(1P)

2. 1on1 digital lifecoaching

Auch hier könnte es sich um eine elektronische Dienstleistung nach Art. 58 I lit. c MwStSystRL handeln, schließlich wird das coaching digital erbracht. Allerdings liegt hier keine Automatisierung vor, im Zentrum steht die vom Menschen erbrachte Leistung des Coachings.(1P) Es handelt sich deshalb um eine reguläre Dienstleistungen s.o., deren Ort sich nach Art. 45 MwStSystRL bestimmt, da H kein Steuerpflichtiger ist. Ort der Leistung ist damit der Sitz der L in den Niederlanden.(1P) Die Leistung wurde von L im Rahmen ihres Unternehmens gegen Entgelt in den Niederlanden erbracht und ist dort steuerbar. (1P)

3. Seminar

Der Ort der Dienstleistung bestimmt sich hier nach Art. 54 MwStSystRL. Bei dem Seminar handelt könnte es sich um Unterricht handeln, bestimmt jedenfalls aber um Unterhaltung oder ähnliche Veranstaltungen. Leistungsort ist der Ort, an dem die Veranstaltung tatsächlich stattfindet, also Köln, Deutschland.(1P) Die Leistung wurde von L im Rahmen ihres Unternehmens gegen Entgelt in Deutschland erbracht und ist dort steuerbar. (1P)

### **(iv) SteuerLLM response (deterministic decoding)**

Die umsatzsteuerlichen Auswirkungen der Umsätze der Spiritcoaching AG (S) für Heribert Gabler (H) im Besteuerungszeitraum 2020 sind wie folgt:

### 1. Einstiegskurs

**Beschreibung:** Digitaler Online-Kurs, bestehend aus aufgezeichneten Lehrvideos und automatisierten, interaktiven Klick-Elementen.

**Steuerbarkeit und Leistungsort:**

- **Steuerbarkeit:** Der Einstiegskurs ist eine auf elektronischem Weg erbrachte sonstige Leistung.

- **Leistungsort:** Gemäß § 3a Abs. 5 Satz 1 UStG ist der Leistungsort für auf elektronischem Weg erbrachte sonstige Leistungen an Nichtunternehmer der Ort, an dem der Leistungsempfänger seinen Wohnsitz hat. Da Heribert in Deutschland wohnt, ist der Leistungsort in Deutschland.

**Steuerpflicht:**

- **Steuerpflicht:** Die Leistung ist steuerpflichtig, da sie in Deutschland steuerbar ist.

**Bemessungsgrundlage:**

- **Bemessungsgrundlage:** Das Entgelt für den Einstiegskurs.

**Steuerentstehung:**

- **Steuerentstehung:** Die Steuer entsteht mit Ablauf des Voranmeldungszeitraums, in dem die Leistung ausgeführt wurde (§ 13 Abs. 1 Nr. 1a UStG).

**Steuerschuldnerschaft:**

- **Steuerschuldnerschaft:** Die Steuerschuldnerschaft liegt bei der Spiritcoaching AG (S), da es sich um eine B2C-Leistung handelt.

**Vorsteuerabzug:**

- **Vorsteuerabzug:** Heribert kann die Vorsteuer nicht abziehen, da er kein Unternehmer ist.

### 2. „1on1 digital lifecoaching“

**Beschreibung:** Persönliche Videokonferenz, in der einer der Gurus vom Sitz in den Niederlanden aus ein persönliches Coaching durchführt.

**Steuerbarkeit und Leistungsort:**

- **Steuerbarkeit:** Das „1on1 digital lifecoaching“ ist eine auf elektronischem Weg erbrachte sonstige Leistung.

- **Leistungsort:** Gemäß § 3a Abs. 5 Satz 1 UStG ist der Leistungsort für auf elektronischem Weg erbrachte sonstige Leistungen an Nichtunternehmer der Ort, an dem der Leistungsempfänger seinen Wohnsitz hat. Da Heribert in Deutschland wohnt, ist der Leistungsort in Deutschland.

**Steuerpflicht:**

- **Steuerpflicht:** Die Leistung ist steuerpflichtig, da sie in Deutschland steuerbar ist.

**Bemessungsgrundlage:**

- **Bemessungsgrundlage:** Das Entgelt für das „1on1 digital lifecoaching“.

**Steuerentstehung:**

- **Steuerentstehung:** Die Steuer entsteht mit Ablauf des Voranmeldungszeitraums, in dem die Leistung ausgeführt wurde (§ 13 Abs. 1 Nr. 1a UStG).

**Steuerschuldnerschaft:**

- **Steuerschuldnerschaft:** Die Steuerschuldnerschaft liegt bei der Spiritcoaching AG (S), da es sich um eine B2C-Leistung handelt.

**Vorsteuerabzug:**

- **Vorsteuerabzug:** Heribert kann die Vorsteuer nicht abziehen, da er kein Unternehmer ist.

### 3. Präsenzveranstaltung in Köln

**Beschreibung:** Seminar in einer großen Kölner Hotelhalle, in der die besten Coaches persönlich anreisen und ihre Erkenntnisse teilen.

**Steuerbarkeit und Leistungsort:**

- **Steuerbarkeit:** Die Präsenzveranstaltung ist eine sonstige Leistung, die nicht auf elektronischem Weg erbracht wird.

- **Leistungsort:** Gemäß § 3a Abs. 3 Nr. 3a UStG ist der Leistungsort für kulturelle, künstlerische, wissenschaftliche, unterrichtende, sportliche, unterhaltende oder ähnliche Leistungen der Ort, an dem die Leistung tatsächlich erbracht wird. Da die Veranstaltung in Köln stattfindet, ist der Leistungsort in Köln.

**Steuerpflicht:**

- **Steuerpflicht:** Die Leistung ist steuerpflichtig, da sie in Deutschland steuerbar ist.

**Bemessungsgrundlage:**

- **Bemessungsgrundlage:** Das Entgelt für die Präsenzveranstaltung.

**Steuerentstehung:**

- **Steuerentstehung:** Die Steuer entsteht mit Ablauf des Voranmeldungszeitraums, in dem die Leistung ausgeführt wurde (§ 13 Abs. 1 Nr. 1a UStG).

**Steuerschuldnerschaft:**

- **Steuerschuldnerschaft:** Die Steuerschuldnerschaft liegt bei der Spiritcoaching AG (S), da es sich um eine B2C-Leistung handelt.

**Vorsteuerabzug:**

- **Vorsteuerabzug:** Heribert kann die Vorsteuer nicht abziehen, da er kein Unternehmer ist.

### Zusammenfassung

- **Einstiegskurs:** Steuerpflichtig in Deutschland, Steuerschuldnerschaft bei S, keine Vorsteuerabzugsmöglichkeit für Heribert.

- **„1on1 digital lifecoaching“:** Steuerpflichtig in Deutschland, Steuerschuldnerschaft bei S, keine Vorsteuerabzugsmöglichkeit für Heribert.

- **Präsenzveranstaltung in Köln:** Steuerpflichtig in Deutschland, Steuerschuldnerschaft bei S, keine Vorsteuerabzugsmöglichkeit für Heribert.

Die Steuer für die steuerpflichtigen Umsätze ist entsprechend zu berechnen und in den jeweiligen Voranmeldungszeiträumen zu berücksichtigen.

### **(iii)+(v) Statement-level decomposition and awarded points**

| i | Reference statement (with m_{q,i}) | Awarded a_{q,i} – adjudicator reasoning |
| --- | --- | --- |
| 1 | Die Spiritcoaching AG ist Steuerpflichtiger i.S.d. Art. 9 I MwStSystRL, da sie eine wirtschaftliche Tätigkeit zur Erzielung von Einnahmen betreibt.  m_{q,1} = 1 P | 0 / 1 P  Der Prüfling hat nicht explizit erwähnt, dass die Spiritcoaching AG eine wirtschaftliche Tätigkeit zur Erzielung von Einnahmen betreibt. |
| 2 | Sie erbringt im Rahmen ihres Unternehmens drei separate Dienstleistungen i.S.d. Art. 24 I MwStSystRL an den H.  m_{q,2} = 1 P | 1 / 1 P  Der Prüfling hat korrekt erkannt, dass die Spiritcoaching AG drei separate Dienstleistungen erbringt. |
| 3 | Der H ins in seiner Rolle als Konsument kein Steuerpflichtiger im Sinne der MwStSystRL.  m_{q,3} = 1 P | 1 / 1 P  Der Prüfling hat korrekt dargestellt, dass Heribert kein Unternehmer ist und somit keinen Vorsteuerabzug hat, was impliziert, dass er kein Steuerpflichtiger im Sinne der MwStSystRL ist. |
| 4 | Es handelt sich um eine elektronisch erbrachte Dienstleistung nach Art. 58 I lit. c MwStSystRL, die an einen nicht-steuerpflichtigen erbracht wurde.  m_{q,4} = 1 P | 1 / 1 P  Der Prüfling hat korrekt erkannt, dass es sich um eine elektronisch erbrachte Dienstleistung handelt, die an einen Nichtunternehmer erbracht wurde. |
| 5 | Der Ort der Dienstleistung ist der Ort der Ansässigkeit/ des Wohnsitzes des H, damit in Nürnberg, Deutschland. Der Umsatz wurde gegen Entgelt erbracht und ist damit in Deutschland steuerbar.(1P)  m_{q,5} = 1 P | 1 / 1 P  Der Prüfling hat korrekt erkannt, dass der Leistungsort für den Einstiegskurs in Deutschland liegt, da der Leistungsempfänger dort wohnt. |
| 6 | Bezüglich des Seminars: Die Leistung wurde von L im Rahmen ihres Unternehmens gegen Entgelt in Deutschland erbracht und ist dort steuerbar. (1P)  m_{q,6} = 1 P | 1 / 1 P  Der Prüfling hat korrekt angegeben, dass die Leistung in Deutschland erbracht und steuerbar ist. |
| 7 | Es handelt sich deshalb um eine reguläre Dienstleistungen s.o., deren Ort sich nach Art. 45 MwStSystRL bestimmt, da H kein Steuerpflichtiger ist. Ort der Leistung ist damit der Sitz der L in den Niederlanden.(1P).  m_{q,7} = 1 P | 0 / 1 P  Der Prüfling hat den Leistungsort für das '1on1 digital lifecoaching' als Deutschland bestimmt, während die Goldantwort den Leistungsort in den Niederlanden sieht. |
| 8 | Auch hier könnte es sich um eine elektronische Dienstleistung nach Art. 58 I lit. c MwStSystRL handeln, schließlich wird das coaching digital erbracht. Allerdings liegt hier keine Automatisierung vor, im Zentrum steht die vom Menschen erbrachte Leistung des Coachings.  m_{q,8} = 1 P | 0 / 1 P  Der Prüfling hat das Coaching als elektronische Dienstleistung klassifiziert, ohne die fehlende Automatisierung zu berücksichtigen. |
| 9 | Die Leistung wurde von L im Rahmen ihres Unternehmens gegen Entgelt in den Niederlanden erbracht und ist dort steuerbar. (1P)  m_{q,9} = 1 P | 0 / 1 P  Der Prüfling hat den Leistungsort für das '1on1 digital lifecoaching' falsch angegeben, da er Deutschland statt den Niederlanden nennt. |
| 10 | Seminar  Der Ort der Dienstleistung bestimmt sich hier nach Art. 54 MwStSystRL. Bei dem Seminar handelt könnte es sich um Unterricht handeln, bestimmt jedenfalls aber um Unterhaltung oder ähnliche Veranstaltungen. Leistungsort ist der Ort, an dem die Veranstaltung tatsächlich stattfindet, also Köln, Deutschland.(1P)  m_{q,10} = 1 P | 1 / 1 P  Der Prüfling hat korrekt erkannt, dass der Leistungsort für das Seminar in Köln ist, da es sich um eine unterhaltende oder ähnliche Veranstaltung handelt. |

Total awarded: Σ a_{q,i} = 6.0 / 10.0 P (60.0 %)

# Supplementary references

1. William G., C. *Sampling Techniques*. (Wiley, 1991).

2. Konietschke, F. & Pauly, M. Bootstrapping and permuting paired t-test type statistics. *Stat Comput* **24**, 283–296 (2014).

3. Dietterich, T. G. Approximate Statistical Tests for Comparing Supervised Classification Learning Algorithms. *Neural Computation* **10**, 1895–1923 (1998).

4. Benjamini, Y. & Hochberg, Y. Controlling the False Discovery Rate: A Practical and Powerful Approach to Multiple Testing. *Journal of the Royal Statistical Society Series B: Statistical Methodology* **57**, 289–300 (1995).

5. Bai, J. *et al.* Qwen technical report. *arXiv preprint arXiv:2309.16609* (2023).

6. Touvron, H. *et al.* LLaMA: Open and Efficient Foundation Language Models. Preprint at http://arxiv.org/abs/2302.13971 (2023).

7. Grattafiori, A. *et al.* The Llama 3 Herd of Models. Preprint at https://doi.org/10.48550/arXiv.2407.21783 (2024).

8. DeepSeek-AI *et al.* DeepSeek-R1: Incentivizing Reasoning Capability in LLMs via Reinforcement Learning. Preprint at https://doi.org/10.48550/arXiv.2501.12948 (2025).
